# Water as a Reactant: DABCO-Catalyzed Hydration of Activated Alkynes for the Synthesis of Divinyl Ethers

**DOI:** 10.1021/acs.joc.4c01815

**Published:** 2024-09-30

**Authors:** Raquel Diana-Rivero, David S. Rivero, Alba García-Martín, Romen Carrillo, David Tejedor

**Affiliations:** Instituto de Productos Naturales y Agrobiología, Consejo Superior de Investigaciones Científicas, Avda. Astrofísico Francisco Sánchez 3, 38 206 La Laguna, Tenerife, Islas Canarias, Spain

## Abstract

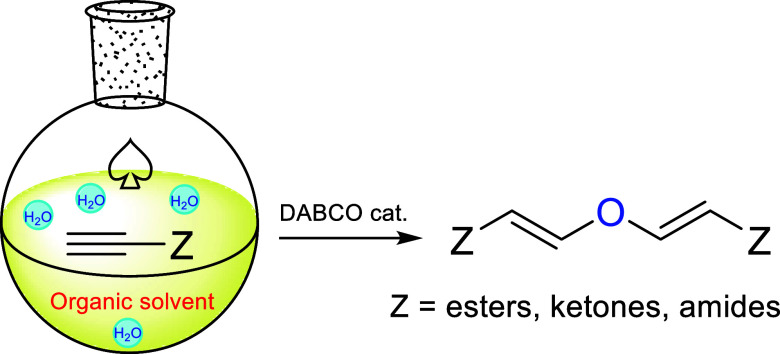

A practical and efficient addition of water to readily available activated alkynes delivering divinyl ethers is reported. The reaction proceeds with full atom economy in a very straightforward experimental procedure. Additionally, of all the tertiary amines studied to catalyze the reaction, the best and most efficient is clearly DABCO (1,4-diazabicyclo[2.2.2]octane). Finally, the solvent choice is crucial for the efficiency of this process and we have found that the reaction is best performed in wet dichloromethane for propiolic esters and alkynones, and in wet acetonitrile for propiolamides.

## Introduction

One can find many applications of water in organic synthesis.^1^ Water as a solvent or water as a useful reagent (as a source of oxygen, a proton or a hydroxyl group) can be found in several examples throughout organic and biological chemistry. The acid- or metal-catalyzed hydration of alkynes affording ketones can be considered one of such examples.^2^ On the other hand, the base catalyzed addition of water onto alkynes has almost no precedents in the literature, and there are only very few occasional examples for the addition onto terminal activated alkynes.^3^ This may not be surprising because water displays a rather low nucleophilicity and it is not known to be a good Michael donor in the reaction with α,β-unsaturated systems. In fact, the rare known double Michael addition of water to acrylonitrile affording 2-cyanoethyl ether needs to be performed in aqueous NaOH.^4^

**Scheme 1:**
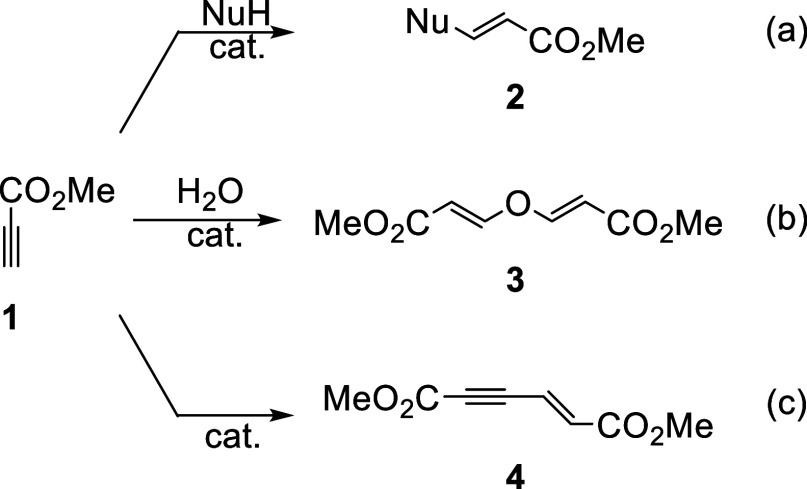
Different Reactivity of Methyl Propiolate under Nucleophilic Catalysis

Our group has an extended experience with the base catalyzed addition of nucleophiles onto activated alkynes.^5^ This powerful click methodology includes the hydroxyl-yne, amino-yne and thiol-yne among others but several additional nucleophiles can be used (Scheme 1a). In some occasions, small amounts of the double addition product **3** can be observed if the reactions are conducted with solvents containing traces of water (Scheme 1b), but this commonly undesired product, which has been shown to have interesting optical properties,^3g^ can be avoided by the used of completely dry solvents. On the other hand, in the absence of suitable nucleophiles, alkyl propiolates dimerize to the corresponding known enyne products **4** (Scheme 1c).^3a,6^

With the current popularity of the click reactions depicted in Scheme 1a, and the increasing number of applications of X-yne click polymerizations,^7^ it is not surprising then that during the course of our own investigation, a very recent example has appeared for the application of the addition of water to activated alkynes.^3g^ Yet, although the stage has been clearly set for further applications, the basis of this reactivity has been completely left ignored and unstudied. As a matter of fact, we believe that the slight current data available is sometimes inaccurate and misleading.^8^ After realizing the potential of this transformation, we herein describe our own findings to shine light about the scope and limitations of this simple, yet powerful organocatalyzed reaction of water and readily available terminal activated alkynes.

## Results and Discussion

**Scheme 2:**
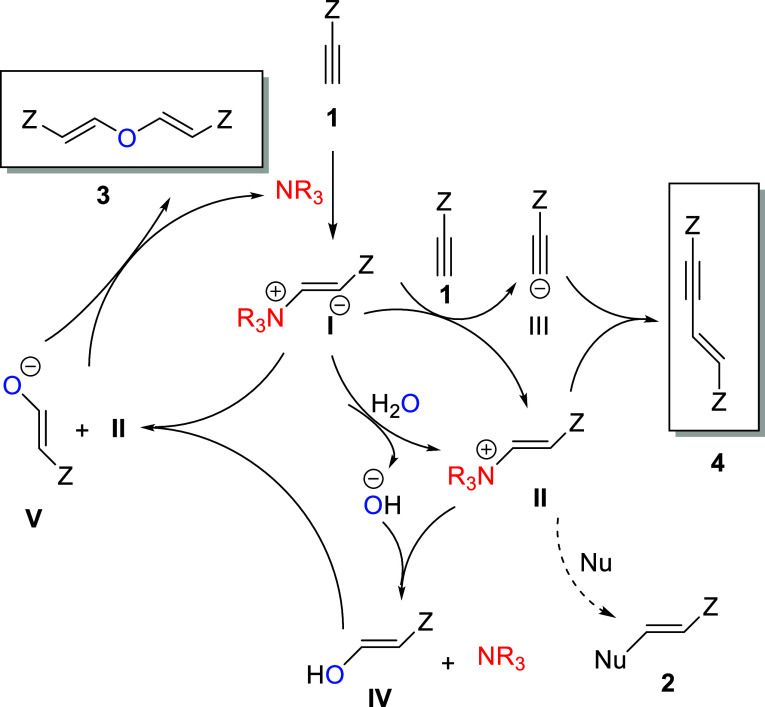
Proposed Mechanistic Cycle for the Formation of Products **2–4**. Z = CO_2_Me or Other Electron-Withdrawing Groups

To fully understand this chemical transformation and to better recognize the steps needed to be taken for the optimization process it was necessary to take a closer look at the proposed mechanistic cycle for the tertiary amine triggered reactivity of activated alkynes shown in Scheme 2.^5a^ Initially, a catalytic amount of a suitable amine adds to the alkyne delivering the zwitterion **I** which is far more basic than the initial species. Most importantly, at this stage the zwitterion is protonated by the most acidic hydrogen present in the reaction medium, that is, a suitable pronucleophile such as an alcohol or a thiol (not shown), water, or in the worst scenario, the activated alkyne itself. Thus, the ammonium **II** forms along with the corresponding anion. It is well studied that appropriate nucleophiles add to this transient ammonium ion to form interesting vinyl products **2**, or even that in their absence, and under typical anhydrous conditions, the enyne dimer of the starting alkyne is the observed product. Interestingly, in the absence of other species more acidic than water itself, intermediate **IV** can form from the addition of the hydroxide ion to the ammonium II, which is followed by the formation of intermediate **V** and more ammonium **II** by an analogous process. Finally, the formation of **3** is a consequence of the coupling of those two intermediates. It should be pointed out that the stereochemistry of the double bonds formed is predominantly or exclusively (*E*), which is consistent with previous reports on nucleophilic additions onto activated alkynes. Furthermore, the assignments were made on the basis of the *J* coupling constants, which were around 12 and 7 Hz for the *E* and *Z* stereochemistries, respectively.

Since we were familiar in our own lab with the formation of trace amounts of usually undesired **3** when adding selected pronucleophiles onto activated alkynes, we now set our goal to maximize its formation. It became obvious that its formation was due to the presence of trace amounts of water in the reaction media from the use of not completely dry solvents or hygroscopic reagents or catalysts. To begin this study, we therefore submitted methyl propiolate to our commonly used reaction conditions (dichloromethane as solvent and DABCO as catalyst) in the absence of any other reagents but using deliberately wet dichloromethane as solvent under different reaction conditions and the results were very promising. At this point we therefore studied different reaction parameters to try to further understand and improve the efficiency of this process. As it can be observed in Table 1, there are several factors that influence the outcome of the reaction and the efficient formation of the now desired product **3a**.

**Table 1:**
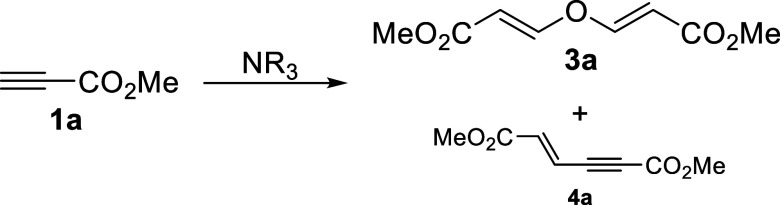
Tertiary Amine-Catalyzed Addition of Water to Methyl Propiolate in Wet Solvents^a^

	NR_3_	mol %	solvent^[^b^]^	[M]	**3a** (%)^[^c^]^**4a** (%)	
1	DABCO	5	DCM	0.2	82	18
2	DABCO	10	DCM	0.2	84	15
3	Et_3_N	10	DCM	0.2	2	
4	NMM	10	DCM	0.2	7	12
5	NMM	50	DCM	0.2	10	24
6	DMAP	10	DCM	0.2		
7	DABCO	10	C_6_H_6_	0.2	39	55
8	DABCO	10	DCE	0.2	59	28
9	DABCO	10	EtOAc	0.2	9	
10	DABCO	10	Et_2_O	0.2	2	
11	DABCO	10	CH_3_CN	0.2	11	34
12	DABCO	10	THF	0.2	6	
13	DABCO	10	H_2_O	0.2	1	2
14	DABCO	10	DCM	0.08	92	1
15	DABCO	5	DCM	0.08	32	3
16	DABCO	2.5	DCM	0.08	1	3
17	DABCO	25	C_6_H_6_	0.08	81^[^d^]^	3
18	DABCO	0	DCM	0.08	0	0

a 2.0 mmol of alkyne, 1 h at room temperature.

b Wet solvent (see also Table S2): DCM, benzene, DCE, EtOAc and Et_2_O saturated with H_2_O prior to use; 5% H_2_O added to dry CH_3_CN and THF prior to use.

c NMR yields using Me_3_SiSiMe_3_ as internal standard.

d A considerable amount of side product methyl (*E*)-3-methoxyacrylate (8%) arising from the reaction of DABCO with the carbonyl group. NMM = *N*-methyl morpholine. DMAP = 4-(dimethylamino)pyridine.

First, while other tertiary amines such as Et_3_N, NMM or DMAP where ineffective, DABCO proved to be by far the best catalyst for this reaction (entries 1–6). This trend is consistent with previous results regarding the addition of alcohols onto activated alkynes and probably arises from its increased nucleophilicity.^5a,9^ Second, the solvent choice also seemed to be very important as the best results were obtained when using dichloromethane. While in benzene the formation of dimer **4a** competes with the formation of the desired divinyl product (entry 7), it seems striking that two solvents which are capable of dissolving more water than dichloromethane are poor solvents for the formation of **3a**. In ethyl acetate or diethyl ether the reaction is very sluggish even after longer reaction times as most of the alkyne remains unreacted (entries 9–10). These latter results suggest that the initial addition of DABCO to methyl propiolate to form intermediate **I** is highly solvent dependent. Acetonitrile and THF, which are miscible with water, or water itself, proved to be also poor solvents for the formation of **3a** (entries 11–13). This may be surprising, especially for THF, since a report claimed otherwise (54% reported yield of **3a**).^3g^ In our hands, we repeatedly obtained only low yields of product **3a** under the reported reaction conditions. For this reason, further experiments were conducted in THF to demonstrate that it is not a solvent of choice for this reaction (see Table S1 in the Supporting Information). As a matter of fact, we could only obtain moderate yields of the desired product when running the reaction in THF doped with 100–400 mol % of H_2_O with respect to **1a**.

Back to using dichloromethane as the solvent, we were able to improve the formation of **3a** by running the reaction under lower concentration (0.08 M instead of 0.2 M, compare entries 2 and 14). This is not surprising because the low solubility of water in dichloromethane determines the amount of water present, and hence, at high concentrations, there is not enough water in the system to participate in the reaction.^10^ Finally, 10% of the catalyst proved to be the optimum amount as the reactivity was hampered by the use of smaller or larger amounts (entries 14–17).

Next, we explored the use of other readily available activated alkynes bearing different electron withdrawing groups, including esters, ketones and amides (Table 2). The reaction starting from alkynes bearing aliphatic esters proved to be very efficient as the desired products are formed in high yields (entries 1–5) while the use of alkynes bearing aromatic esters gave moderate yields (60–65%) due to the formation of undesired sideproduct **5** (entries 6–7, see also Scheme S1).

**Table 2:**
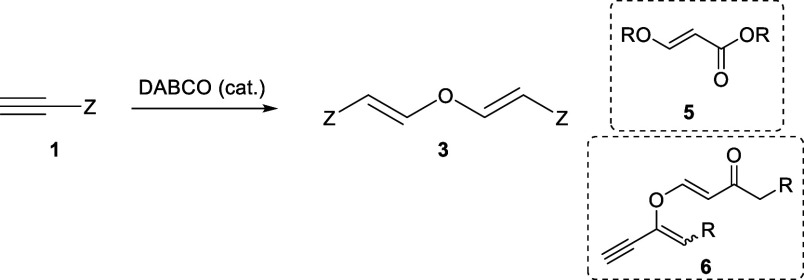

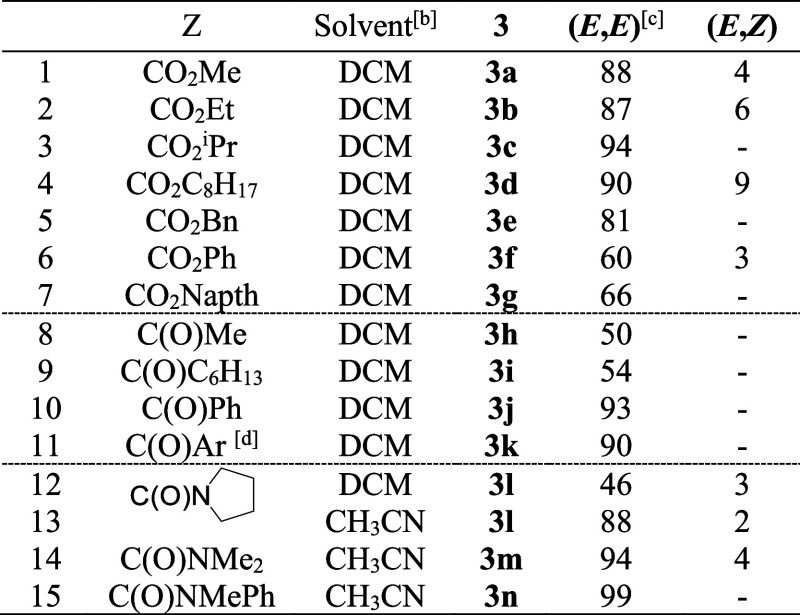
DABCO-Catalyzed Addition of Water to Activated Alkynes in Wet Solvents^a^^,^^b^^,^^c^^,^^d^

a 2.0 mmol of alkyne, 10 mol % of DABCO and 1 h at room temperature for entries 1–11, and 25 mol % and 5 h at room temperature for entries 12–15.

b 25 mL of DCM saturated with H_2_O prior to use or 12 mL of CH_3_CN and 0.5 mL of H_2_O.

c Isolated yields.

d Ar = 3,4,5-trimethoxyphenyl.

Alkynones were affected by the presence of enolizable hydrogens. Thus, whereas aromatic alkynones **1j–k** gave excellent yields of **3j** and **3k** (entries 10–11), aliphatic alkynones **1h**, **i** gave the corresponding desired products in moderate yields (entries 8–9). In these cases the formation of undesired sideproduct **6** was responsible for the decrease in the efficiency (see Scheme S2).

Interestingly, although propiolamides are less reactive, we were able to obtain the desired addition products nonetheless with some important changes in the reaction conditions. They are worse acceptors toward the 1,4-nucleophilic addition of the catalyst, less reactive at the carbonyl and less acidic which means that they will not only react slower but that the formation of sideproducts such as **4** or **5** will be hampered. In these cases, the reactions were more efficient in acetonitrile doped with 13 equiv of water and increasing the amount of DABCO to 25 mol % and the time to 6 h. Thus, tertiary propiolamides **1l–n** afforded the corresponding products in excellent yields (entries 12–15).

It should be highlighted at this point that the scalability of the process is straightforward and that entry 4 was conducted on a gram scale (6.0 mmol, 1.09 g) and the result was maintained.

With regard to the stability of divinyl products, those derived from propiolic esters and propiolamides where quite stable and no special care was taken during the isolation process. On the other hand, products **3h–k** derived from alkynones are acid-sensitive as they decompose even in slightly acidic deuterated chloroform or prolonged exposure to silica gel. NMR spectra of these compounds were recorded in either deuterated benzene or deuterated chloroform pretreated with NaOH pellets to prevent problems with the residual acid content.

**Scheme 3:**
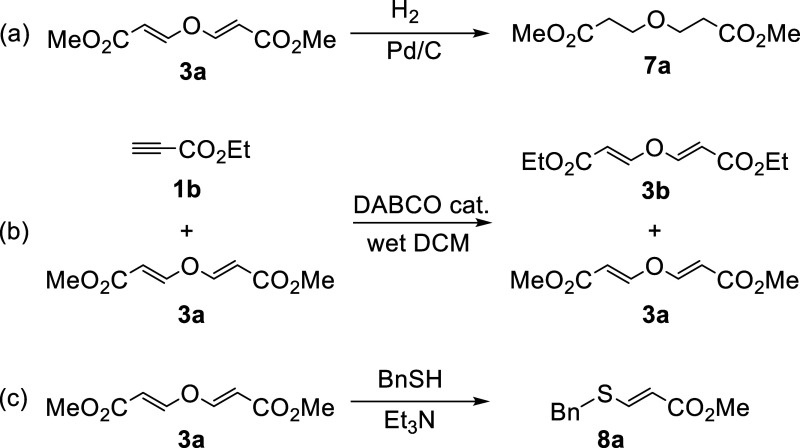
(a) Hydrogenation of **3a**; (b) Irreversibility Experiment; (c) Degradation of **3a** with a Thiolate

In additional experiments, divinyl compounds can be submitted to typical hydrogenation conditions to obtain, for example, the saturated ether **7a** in very high yields (Scheme 3a).

Interestingly, the dynamic nature of the addition reaction was discarded as it seems to be irreversible. Mixing ethyl propiolate with an equimolar amount of product **3a** derived from methyl propiolate in the presence of catalytic amounts of DABCO in wet dichloromethane delivered exclusively the symmetrical product **3b** with no signs of a mixed divinyl compound containing different esters (Scheme 3b). However, hydrated compounds can be selectively degraded by the action of a good nucleophile: the model compound **3a** proved to be reactive in the presence of a thiolate to deliver a vinyl sulfide **8a** (Scheme 3c). This property is highly relevant, because any small compound or polymer made with the DABCO-catalyzed reaction herein described could be eventually degraded upon the application of the right chemical stimulus. Such control is quite useful for the development of responsive systems^11^ and/or degradable polymers.^12^

## Conclusion

In summary, herein we have reported the practical and efficient addition of water to readily available activated alkynes delivering divinyl ethers. The reaction proceeds with full atom economy, while it is catalyzed by DABCO and best performed in wet dichloromethane for propiolic esters and alkynones, and in wet acetonitrile for propiolamides. Since some divinyl ethers have been previously shown to present interesting optical properties, and we have gained access to new compounds which were not previously known (those containing amide groups), this research opens up the venue for further study in their properties. Additionally, we proved that this kind of divinyl ether compounds can be selectively degraded, and therefore we hope that this study triggers new research in X-yne polymerizations and responsive molecular systems.

## Experimental Section

### General Remarks

All reagents from commercial suppliers were used without further purification. All solvents were freshly distilled before use from appropriate drying agents. Analytical TLCs were performed with silica gel 60 F254 plates. Visualization was accomplished by naked eye, or by UV light or vanillin with acetic and sulfuric acid in ethanol with heating. Column chromatography was carried out using silica gel 60 (230–400 mesh ASTM). ^1^H NMR spectra were recorded at 500 and 400 MHz, ^13^C NMR spectra were recorded at 125 and 100 MHz. Data were reported as follows: chemical shift, multiplicity (s = singlet, d = doublet, t = triplet, q = quartet, dd = double doublet, m = multiplet and br = broad), coupling constant (*J* values) in Hz and integration. High resolution mass spectra (HRMS) were measured by ESI method with an Agilent LC-Q-TOF-MS 6520 spectrometer. Alkynes **1a**, **1b** and **1h** are commercially available. The rest of the alkynes used in this work are known and were synthesized following established literature methods.^3e^

### General Procedure for the Reaction of Activated Alkynes in Wet Dichloromethane Catalyzed by DABCO

To an oven-dried round-bottom flask containing the corresponding alkyne (2.00 mmol, 0.08 M), was added wet dichloromethane saturated with H_2_O (25 mL, previously shaken off with H_2_O in a separatory funnel) and finally DABCO (22.4 mg, 0.20 mmol). The reaction was stirred for 1 h at room temperature although the reaction seemed completed by TLC analysis within minutes. The resulting mixture was then filtrated over anhydrous Na_2_SO_4_ and the solvent was evaporated under reduced pressure to get a crude solid which was then subjected to Flash chromatography (appropriate mixtures of ethyl acetate: hexane, unless mentioned otherwise) to get the desired products (**3a–3k**).

### General Procedure for the Reaction of Propiolamides in Wet Acetonitrile Catalyzed by DABCO

To an oven-dried round-bottom flask containing the corresponding propiolamide (1.00 mmol, 0.17 M), was added dry acetonitrile (6 mL), H_2_O (0.25 mL) and finally DABCO (28 mg, 0.25 mmol). The reaction was stirred for 5 h at room temperature. The resulting mixture was then filtrated over anhydrous Na_2_SO_4_ and the solvent was evaporated under reduced pressure to get a crude solid which was then subjected to Flash chromatography (1–5% methanol: dichloromethane) to get the desired products (**3l–3n**).

#### Dimethyl 3,3′-Oxy(2*E*,2′*E*)-diacrylate **3a** (*E*,*E*)

163.8 mg, 88% of a white crystalline solid purified by column chromatography (ethyl acetate/hexanes = 1:4). ^1^H NMR (CDCl_3_, 400 MHz, δ): 7.57 (d, 2H, *J* = 12.1 Hz), 5.65 (d, 2H, *J* = 12.1 Hz), 3.73 (s, 6H). ^13^C{^1^H} NMR (CDCl_3_, 100 MHz): δ = 166.4, 157.4, 103.9, 51.6 ppm. Melting point 158–159 °C. Data in full accordance with that reported in the literature.^3g^

#### Methyl (*Z*)-3-(((*E*)-3-Methoxy-3-oxoprop-1-en-1-yl)oxy)acrylate **3a** (*E*,*Z*)

7.5 mg, 4% of a crystalline white solid purified by column chromatography (ethyl acetate/hexanes = 1:4). ^1^H NMR (CDCl_3_, 500 MHz, δ):7.56 (d, 1H, *J* = 12.2 Hz), 6.69 (d, 1H, *J* = 6.9 Hz), 5.71 (d, 1H, *J* = 12.2 Hz), 5.20 (d, 1H, *J* = 6.9 Hz), 3.72 (s, 6H). ^13^C{^1^H} NMR (CDCl_3_, 126 MHz): δ = 166.7, 164.1, 158.4, 152.3, 103.3, 101,8, 51.6, 51.5 ppm. Melting point 108–109 °C. Data in full accordance with that reported in the literature.^3g^

#### Diethyl 3,3′-Oxy(2*E*,2′*E*)-diacrylate **3b** (*E*,*E*)

370.4 mg, 87% of a white crystalline solid purified by column chromatography (ethyl acetate/hexanes = 1:4). ^1^H NMR (CDCl_3_, 400 MHz, δ):7.56 (d, 2H, *J* = 12.2 Hz), 5.63 (d, 2H, *J* = 12.2 Hz), 4.18 (q, 4H, *J* = 7.1 Hz), 1.27 (t, 6H, *J* = 7.1 Hz). ^13^C{^1^H} NMR (CDCl_3_, 100 MHz): δ = 166.0, 157.2, 104.3, 60.5, 14.2 ppm. Melting point 113–114 °C. Data in full accordance with that reported in the literature.^3c^

#### Ethyl (*Z*)-3-(((*E*)-3-Ethoxy-3-oxoprop-1-en-1-yl)oxy)acrylate **3b** (*E*,*Z*)

26.3 mg, 6% of a white crystalline solid purified by column chromatography (ethyl acetate/hexanes = 1:4). ^1^H NMR (CDCl_3_, 400 MHz, δ):7.55 (d, 1H, *J* = 12.2 Hz), 6.69 (d, 1H, *J* = 6.9 Hz), 5.67 (d, 1H, *J* = 12.2 Hz), 5.17 (d, 1H, *J* = 6.9 Hz), 4.18 (q, 4H, *J* = 7.1 Hz), 1.27 (t, 3H, *J* = 7.1 Hz), 1.26 (t, 3H, *J* = 7.1 Hz). ^13^C{^1^H} NMR (CDCl_3_, 100 MHz): δ = 166.3, 163.7, 158.3, 152.2, 103.6, 102.2, 60.4, 60.3, 14.2, 14.1 ppm. Melting point 50–52 °C. HRMS (TOF MS AP^+^): *m*/*z* [M + H]^+^ calcd for C_10_H_15_O_5_, 215.0919; found, 215.0921.

#### Diisopropyl 3,3′-oxy(2*E*,2′*E*)-diacrylate **3c** (*E*,*E*)

211.8 mg, 94% of a white crystalline solid purified by column chromatography (ethyl acetate/hexanes = 1:5). ^1^H NMR (CDCl_3_, 400 MHz, δ):7.53 (d, 2H, *J* = 12.2 Hz), 5.60 (d, 2H, *J* = 12.2 Hz), 5.02–5.09 (m, 2H), 1.24 (d, 12H, *J* = 6.3 Hz). ^13^C{^1^H} NMR (CDCl_3_, 100 MHz): δ = 165.6, 157.1, 104.6, 67.9, 21.8 ppm. Melting point 120–121 °C. HRMS (TOF MS AP^–^): *m*/*z* [M – H]^−^ calcd for C_12_H_17_O_5_, 241.1076; found, 241.1086.

#### Dioctyl 3,3′-Oxy(2*E*,2′*E*)-diacrylate **3d** (*E*,*E*)

1.037 g, 90% of a white crystalline solid purified by column chromatography (ethyl acetate/hexanes = 1:9). ^1^H NMR (CDCl_3_, 400 MHz, δ):7.55 (d, 2H, *J* = 12.2 Hz), 5.64 (d, 2H, *J* = 12.2 Hz), 4.12 (t, 4H, *J* = 6.7 Hz), 1.60–1.67 (m, 4H), 1.25–1.37 (m, 20H), 0.87 (t, 6H, *J* = 7.0 Hz). ^13^C{^1^H} NMR (CDCl_3_, 100 MHz): δ = 166.2, 157.2, 104.3, 64.7, 31.8, 29.2, 29.1, 28.6, 25.9, 22.6, 14.1 ppm. Melting point 36–37 °C. HRMS (TOF MS AP^+^): *m*/*z* [M + H]^+^ calcd for C_22_H_39_O_5_, 383.2797; found, 383.2800.

#### Octyl (*Z*)-3-(((*E*)-3-(Octyloxy)-3-oxoprop-1-en-1-yl)oxy)acrylate **3d** (*E*,*Z*)

102.6 mg, 9% of a colorless oil purified by column chromatography (ethyl acetate/hexanes = 1:9). ^1^H NMR (CDCl_3_, 400 MHz, δ):7.55 (d, 1H, *J* = 12.2 Hz), 6.69 (d, 1H, *J* = 6.9 Hz), 5.67 (d, 1H, *J* = 12.2 Hz), 5.18 (d, 1H, *J* = 6.9 Hz), 4.11 (t, 4H, *J* = 6.7 Hz), 1.60–1.67 (m, 4H), 1.22–1.36 (m, 20H), 0.86 (t, 6H, *J* = 7.0 Hz). ^13^C{^1^H} NMR (CDCl_3_, 100 MHz): δ = 166.4, 163.9, 158.3, 152.1, 103.6, 102.3, 64.7, 64.5, 31.8 (2C), 29.2 (4C), 28.61, 28.57, 25.92, 25.89, 22.6 (2C), 14.1 (2C) ppm. HRMS (TOF MS ES^+^): *m*/*z* [M + Na]^+^ calcd for C_22_H_38_O_5_Na, 405.2617; found, 405.2618.

#### (2*E*,2′*E*)-3,3′-Oxy-dibenzyldiacrylate **3e** (*E*,*E*)

273 mg, 81% of a white amorphous solid purified by column chromatography (ethyl acetate/hexanes = 1:4). ^1^H NMR (CDCl_3_, 400 MHz, δ):7.59 (d, 2H, *J* = 12.2 Hz), 7.37 (s, 10H), 5.70 (d, 2H, *J* = 12.1 Hz), 5.19 (s, 2H). ^13^C{^1^H} NMR (CDCl_3_, 100 MHz): δ = 166.0, 157.7, 135.9, 128.8, 128.5, 128.4, 104.3, 77.5, 77.2, 76.8, 66.5 ppm. HRMS (TOF MS ES^+^): *m*/*z* [M + Na]^+^ calcd for C_20_H_18_O_5_Na, 361.1052; found, 361.1048.

#### Diphenyl 3,3′-Oxy(2*E*,2′*E*)-diacrylate **3f** (*E*,*E*)

Twenty mol % DABCO used in the reaction. 185.9 mg, 60% of a white amorphous solid purified by column chromatography (ethyl acetate/hexanes = 1:4). ^1^H NMR (CDCl_3_, 400 MHz, δ):7.81 (d, 2H, *J* = 12.1 Hz), 7.40 (t, 4H, *J* = 7.8 Hz), 7.24 (t, 2H, *J* = 7.4 Hz), 7.12 (d, 4H, *J* = 7.8 Hz), 5.91 (d, 2H, *J* = 12.1 Hz). ^13^C{^1^H} NMR (CDCl_3_, 100 MHz): δ = 164.4 (2C), 158.5 (2C), 150.3 (2C), 129.4 (4C), 125.9 (2C), 121.5 (4C), 104.2 (2C) ppm. HRMS (TOF MS ES^+^): *m*/*z* [M + Na]^+^ calcd for C_18_H_14_O_5_Na, 333.0739; found, 333.0740.

#### Phenyl (*Z*)-3-(((*E*)-3-oxo-3-phenoxyprop-1-en-1-yl)oxy)acrylate **3f** (*E*,*Z*)

9.5 mg, 3% of a colorless oil purified by column chromatography (ethyl acetate/hexanes = 1:4). ^1^H NMR (CDCl_3_, 400 MHz, δ):7.79 (d, 1H, *J* = 12.1 Hz), 7.37–7.42 (m, 4H), 7.23–7.26 (m, 2H), 7.11–7.16 (m, 4H), 6.92 (d, 1H, *J* = 6.8 Hz), 5.93 (d, 1H, *J* = 12.1 Hz), 5.47 (d, 1H, *J* = 6.8 Hz). ^13^C{^1^H} NMR (CDCl_3_, 100 MHz): δ = 164.7, 161.8, 159.5, 153.7, 150.4 (2C), 129.42 (2C), 129.38 (2C), 125.90, 125.86, 121.56 (4C), 103.4, 101.7 ppm. HRMS (TOF MS ES^+^): *m*/*z* [M + Na]^+^ calcd for C_18_H_14_O_5_Na, 333.0739; found, 333.0742.

#### Di(naphthalen-1-yl) 3,3′-Oxy(2*E*,2′*E*)-diacrylate **3g** (*E*,*E*)

0.50 mmol of alkyne in 25 mL of wet dichloromethane, 20 mol % of DABCO. 135.3 mg, 66% of an off-white crystalline solid purified by column chromatography (ethyl acetate/hexanes = 1:4). ^1^H NMR (CDCl_3_, 400 MHz, δ):7.96 (d, 2H, *J* = 12.1 Hz), 7.86–7.90 (m, 4H), 7.77 (d, 2H, *J* = 8.3 Hz), 7.51–7.53 (m, 4H), 7.48 (t, 2H, *J* = 7.9 Hz), 7.29 (d, 2H, *J* = 7.4 Hz), 6.11 (d, 2H, *J* = 12.1 Hz). ^13^C{^1^H} NMR (CDCl_3_, 100 MHz): δ = 164.5, 158.8, 146.2, 134.7, 128.1, 126.8, 126.6, 126.5, 126.2, 125.4, 121.1, 118.1, 103.9 ppm. Melting point 178–179 °C. HRMS (TOF MS ES^+^): *m*/*z* [M + Na]^+^ calcd for C_26_H_18_O_5_Na, 433.1052; found, 433.1060.

#### (3*E*,3′*E*)-4,4′-Oxybis(but-3-en-2-one) **3h** (*E*,*E*)

83.9 mg, 50% of a white amorphous solid purified by column chromatography (ethyl acetate/hexanes = 1:1). ^1^H NMR (CDCl_3_, 400 MHz, δ):7.52 (d, 2H, *J* = 12.2 Hz), 5.99 (d, 2H, *J* = 12.2 Hz), 2.23 (m, 6H). ^13^C{^1^H} NMR (CDCl_3_, 100 MHz): δ = 196.4, 156.8, 113.1, 28.7 ppm. Data in full accordance with that reported in the literature.^3g^

#### (1*E*,1′*E*)-1,1′-Oxybis(oct-1-en-3-one) **3i** (*E*,*E*)

143.8 mg, 54% of a white amorphous solid purified by column chromatography (ethyl acetate/hexanes = 1:4). ^1^H NMR (CDCl_3_, 400 MHz, δ):7.20 (d, 2H, *J* = 12.2 Hz), 5.80 (d, 2H, *J* = 12.2 Hz), 2.15 (t, 4H, *J* = 7.3 Hz), 1.61–1.69 (m, 4H), 1.22–1.35 (m, 8H), 0.96 (t, 6H, *J* = 6.8 Hz). ^13^C{^1^H} NMR (CDCl_3_, 100 MHz): δ = 199.2, 156.3, 111.8, 42.1, 31.3, 23.8, 22.4, 13.8 ppm. HRMS (TOF MS ES^+^): *m*/*z* [M + Na]^+^ calcd for C_16_H_26_O_3_Na, 289.1780; found, 289.1783.

#### (2*E*,2′*E*)-3,3′-Oxybis(1-phenylprop-2-en-1-one) **3j** (*E*,*E*)

Purified by flash chromatography using 1% ethyl acetate in dichloromethane as eluent. 258.8 mg, 93% of a white amorphous solid: ^1^H NMR (C_6_D_6_, 400 MHz, δ):7.90 (t, 4H, *J* = 7.8 Hz), 7.57 (d, 2H, *J* = 11.7 Hz), 7.22 (d, 4H, *J* = 7.8 Hz), 7.16 (t, 2H, *J* = 7.4 Hz), 6.62 (d, 2H, *J* = 11.7 Hz). ^13^C{^1^H} NMR (C_6_D_6_, 100 MHz): δ = 188.3, 158.8, 138.3, 132.7, 128.70, 128.4, 107.8 ppm. HRMS (TOF MS ES^+^): *m*/*z* [M + Na]^+^ calcd for C_18_H_14_O_3_Na, 301.0841; found, 301.0842.

#### (2*E*,2′*E*)-3,3′-Oxybis(1-(3,4,5-trimethoxyphenyl)prop-2-en-1-one) **3k** (*E*,*E*)

Purified by flash chromatography using 1% ethyl acetate in dichloromethane as eluent. 61.8 mg, 90% of a white crystalline solid: ^1^H NMR (CDCl_3_, 400 MHz, δ):7.82 (d, 2H, *J* = 11.7 Hz), 7.18 (s, 4H), 6.81 (d, 2H, *J* = 11.7 Hz), 3.92 (s, 18H). ^13^C{^1^H} NMR (CDCl_3_, 100 MHz): δ = 188.1, 158.4, 153.2, 142.8, 132.9, 107.9, 105.7, 61.0, 56.4 ppm. Melting point 169–170 °C. HRMS (TOF MS ES^+^): *m*/*z* [M + H]^+^ calcd for C_24_H_27_O_9_ 459.1655; found, 459.1636.

#### (2*E*,2′*E*)-3,3′-Oxybis(1-(pyrrolidin-1-yl)prop-2-en-1-one). **3l** (*E*,*E*)

117 mg, 88% of a white crystalline solid purified by column chromatography (methanol/dichloromethane = 1:25). ^1^H NMR (CDCl_3_, 400 MHz, δ):7.61 (d, 2H, *J* = 11.7 Hz), 5.90 (d, 2H, *J* = 11.7 Hz), 3.47 (br s, 8H), 1.90 (br s, 8H). ^13^C{^1^H} NMR (CDCl_3_, 126 MHz): δ = 163.9, 155.9, 103.8, 46.1, 25.8, 24.6 ppm. HRMS (TOF MS ES^+^): *m*/*z* [M + Na]^+^ calcd for C_14_H_20_N_2_O_3_Na, 287.1372; found, 287.1370. Melting point 178–179 °C.

#### (*Z*)-3-(((*E*)-3-Oxo-3-(pyrrolidin-1-yl)prop-1-en-1-yl)oxy)-1-(pyrrolidin-1-yl)prop-2-en-1-one **3l** (*E*,*Z*)

3.2 mg, 2.4% of a white crystalline solid purified by column chromatography (methanol/dichloromethane = 1:25). ^1^H NMR (CDCl_3_, 400 MHz, δ):7.58 (d, 1H, *J* = 11.6 Hz), 6.57 (d, 1H, *J* = 7.0 Hz), 5.97 (d, 1H, *J* = 11.6 Hz), 5.26 (d, 1H, *J* = 7.0 Hz) 3.47 (br s, 8H), 1.89 (br s, 8H). ^13^C{^1^H} NMR (CDCl_3_, 100 MHz): δ = 164.4, 162.9, 157.3, 148.8, 103.5, 103.3, 46.5, 25.9, 24.2 ppm. HRMS (TOF MS ES^+^): *m*/*z* [M + Na]^+^ calcd for C_14_H_20_N_2_O_3_Na, 287.1372; found, 287.1369. Melting point 158–159 °C.

#### (2*E*,2′*E*)-3,3′-Oxybis(*N*,*N*-dimethylacrylamide) **3m** (*E*,*E*)

99.8 mg, 94% of a white crystalline solid purified by column chromatography (methanol/dichloromethane = 1:25). ^1^H NMR (CDCl_3_, 400 MHz, δ):7.58 (d, 2H, *J* = 11.5 Hz), 6.06 (d, 2H, *J* = 11.5 Hz), 2.96 (s, 6H), 3.00 (s, 6H). ^13^C{^1^H} NMR (CDCl_3_, 100 MHz): δ = 165.6, 156.4, 102.4, 37.2, 35.4 ppm. HRMS (TOF MS ES^+^): *m*/*z* [M + Na]^+^ calcd for C_10_H_16_N_2_O_3_Na 235.1059; found, 235.1058. Melting point 152–153 °C.

#### (*Z*)-3-(((*E*)-3-(Dimethylamino)-3-oxoprop-1-en-1-yl)oxy)-*N*,*N*-dimethylacrylamide **3m** (*E*,*Z*)

3.7 mg, 3.5% of a white solid purified by column chromatography (methanol/dichloromethane = 1:25). ^1^H NMR (CDCl_3_, 400 MHz, δ):7.58 (d, 1H, *J* = 11.5 Hz), 6.54 (d, 1H, *J* = 6.9 Hz), 6.06 (d, 1H, *J* = 11.5 Hz), 5.29 (d, 1H, *J* = 6.9 Hz), 3.00 (s, 12H). ^13^C{^1^H} NMR (CDCl_3_, 100 MHz): δ = 166.0, 164.8, 157.3, 147.2, 103.5, 101.8, 37.8, 37.3, 35.5, 34.7 ppm. HRMS (TOF MS ES^+^): *m*/*z* [M + Na]^+^ calcd for C_10_H_16_N_2_O_3_Na, 235.1059; found, 235.1060.

#### (2*E*,2′*E*)-3,3′-Oxybis(*N*-methyl-*N*-phenylacrylamide) **3n** (*E*,*E*)

179.8 mg, 99% of a white amorphous solid purified by column chromatography (methanol/dichloromethane = 1:25). ^1^H NMR (CDCl_3_, 400 MHz, δ):7.50 (d, 2H, *J* = 11.6 Hz), 7.36–7.40 (m, 4H), 7.28–7.32 (m, 2H), 7.13–7.14 (m, 4H), 5.48 (d, 2H, *J* = 11.6 Hz), 3.31 (s, 6H). ^13^C{^1^H} NMR (CDCl_3_, 100 MHz): δ = 165.3, 156.0, 143.3, 129.6, 127.6, 127.2, 103.8, 37.1 ppm. HRMS (TOF MS ES^+^): *m*/*z* [M + Na]^+^ calcd for C_20_H_20_N_2_O_3_Na, 359.1372; found, 359.1373.

#### Dimethyl (*E*)-Hex-2-en-4-ynedioate **4a**

Isolated in variable amounts depending the reaction conditions (see Table 1). White crystalline solid purified by column chromatography (ethyl acetate/hexanes = 1:4). ^1^H NMR (CDCl_3_, 400 MHz, δ):6.77 (d, 1H, *J* = 16.0 Hz), 6.46 (d, 1H, *J* = 16.0 Hz), 3.81 (s, 3H), 3.78 (s, 3H). ^13^C{^1^H} NMR (CDCl_3_, 100 MHz): δ = 165.1, 153.5, 135.0, 121.7, 86.7, 81.8, 53.1, 52.3 ppm. Melting point 59–60 °C. Data in full accordance with that reported in the literature.^13^

#### Hydrogenation of **3a**

**3a** (100.0 mg, 0.537 mmol) was dissolved in EtOAc (10 mL). 10% Pd/C (5 mol %) was added, and the mixture was stirred at room temperature overnight under hydrogen. After filtration through Celite, the solvent was removed under reduced pressure to give a residue which was purified by flash chromatography (silica gel, 20:80 ethyl acetate/hexane) to afford **7a** (99.4 mg, 97% yield).

#### Dimethyl 3,3′-Oxidipropionate **7a**

99.4 mg, (97%) of a colorless oil purified by column chromatography (ethyl acetate/hexanes = 1:4). ^1^H NMR (CDCl_3_, 400 MHz, δ):3.71 (t, 4H, *J* = 6.4 Hz), 3.67 (s, 6H), 2.55 (t, 4H, *J* = 6.4 Hz). ^13^C{^1^H} NMR (CDCl_3_, 100 MHz): δ = 171.9, 66.2, 51.6, 34.7 ppm. Data in full accordance with that reported in the literature.^14^

#### Reaction of **3a** with Benzyl Mercaptan

To an oven-dried round-bottom flask containing **3a** (1.00 mmol), was added acetonitrile (10 mL) and dichloromethatne (3 mL) followed by benzyl mercaptan (0.23 mL, 2.0 mmol) and Et_3_N (0.35 mL, 2.5 mmol). The reaction was stirred overnight at room temperature. The solvents were evaporated under reduced pressure and the crude mixture was then subjected to flash chromatography (20:80 ethyl acetate/hexane) to give 191.7 mg (0.92 mmol) of **8a** (92%).

#### Methyl (*E*)-3-(Benzylthio)acrylate **8a**

191.7 mg (92%) of a white amorphous solid purified by column chromatography (dichloromethane/hexanes = 1:1): ^1^H NMR (CDCl_3_, 400 MHz, δ):7.70 (d, 1H, *J* = 15.1 Hz), 7.28–7.34 (m, 5H), 5.81 (d, 1H, *J* = 15.1 Hz), 4.01 (s, 2H), 3.70 (s, 3H). ^13^C{^1^H} NMR (CDCl_3_, 100 MHz): δ = 165.6, 146.3, 135.4, 128.8, 128.8, 127.8, 114.0, 51.5, 36.6 ppm. Data in full accordance with that reported in the literature.^15^

## Data Availability

The data underlying this study are available in the published article and its Supporting Information

## Supporting Information Available

^1^H and ^13^C NMR spectra of all products (PDF)
