# Supplementary material for: Water as a Reactant: DABCO-Catalyzed Hydration of Activated Alkynes for the Synthesis of Divinyl Ethers
Source: J Org Chem. 2024 Sep 30;89(20):15068–74. doi: 10.1021/acs.joc.4c01815 (PMC11494655; doi:10.1021/acs.joc.4c01815)
Supplement: Supplementary file 1 — jo4c01815_si_001.pdf [file jo4c01815_si_001.pdf]

SUPPORTING INFORMATION

Water as a Reactant: DABCO-catalyzed Hydration of Activated Alkynes for the Synthesis of Divinyl Ethers

Raquel Diana-Rivero, David S. Rivero, Alba García-Martín, Romen Carrillo* and David Tejedor*

Instituto de Productos Naturales y Agrobiología, Consejo Superior de Investigaciones Científicas, Avda. Astrofísico Francisco Sánchez 3, 38 206 La Laguna, Tenerife, Islas Canarias, Spain.

E-mail: rcarrillo@ipna.csic.es; dtejedor@ipna.csic.es

Table of contents

1. General Information S2

2. Table S1. DABCO-catalyzed addition of water to methyl propiolate in THF/H_2O S2

3. Table S2. Solubility of water in selected organic solvents S2

4. Mechanistic Schemes S3

5. ^{1}H NMR and ^{13}C NMR Spectra S4-S26

General Information

All reagents from commercial suppliers were used without further purification. All solvents were freshly distilled before use from appropriate drying agents. Analytical TLCs were performed with silica gel 60 F254 plates. Visualization was accomplished by naked eye, or by UV light or vanillin with acetic and sulfuric acid in ethanol with heating. Column chromatography was carried out using silica gel 60 (230-400 mesh ASTM). ^{1}H NMR spectra were recorded at 500 MHz and 400MHz, ^{13}C NMR spectra were recorded at 125 MHz and 100 MHz. NaOH lentils were added to $CDCl_3$ to prevent problems with the residual acid content. High resolution mass spectra (HRMS) were measured by ESI method with an Agilent LC-Q-TOF-MS 6520 spectrometer.

1. **Table S1.** DABCO-catalyzed addition of water to methyl propiolate in THF/H_2O.[a]

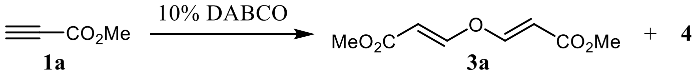

	**1a** (mmoL)	THF (mL)[c]	H_2O	**3a**(%)[d]	**4**(%)
1[b]	5.0	1	5 mL	6	-
2	2.0	1	5 mL	2	-
3	2.0	10	0.5 mL	6	-
4	2.0	10	40 mg	54	10
5	2.0	10	76 mg	68	3
6	2.0	10	150 mg	57	1

[a)]1h at room temperature. NMR yields using $Me_3SiSiMe_3$ as internal standard. [b)] 24 h Exact conditions reported in reference 3g. 54% of product **3a** is reported. [c)] The use of distilled or dry THF from a bottle from a commercial supplier was compared and does not alter significantly the outcome of the reaction. [d)]Predominantly or exclusively (*E*,*E*).

2. **Table S2.** Solubility of water in selected organic solvents.

Entry	Solvent	Solubility (w/w)
1	Dichloromethane	0.24%
2	Dichloroethane	0.15%
3	Benzene	0.063%
4	Ethyl acetate	3.3%
5	Diethyl ether	1.26%
6	Acetonitrile	miscible
7	Tetrahydrofuran	miscible

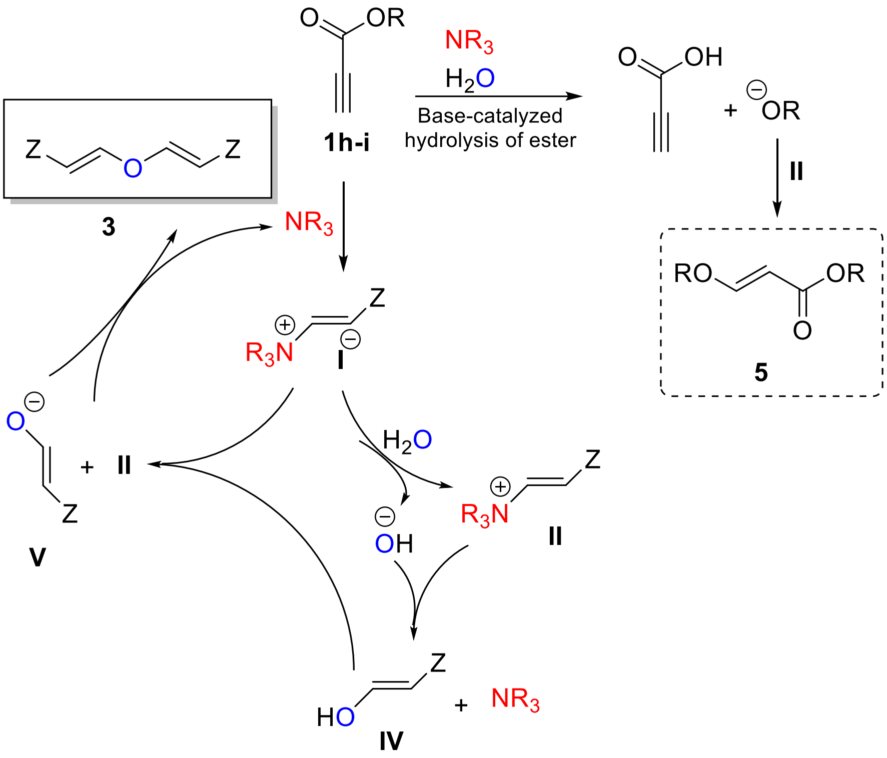

Scheme S1. Mechanistic proposal for the formation of products **3** or **5** when the initial alkyne bears an ester functionality. Z = CO_2R

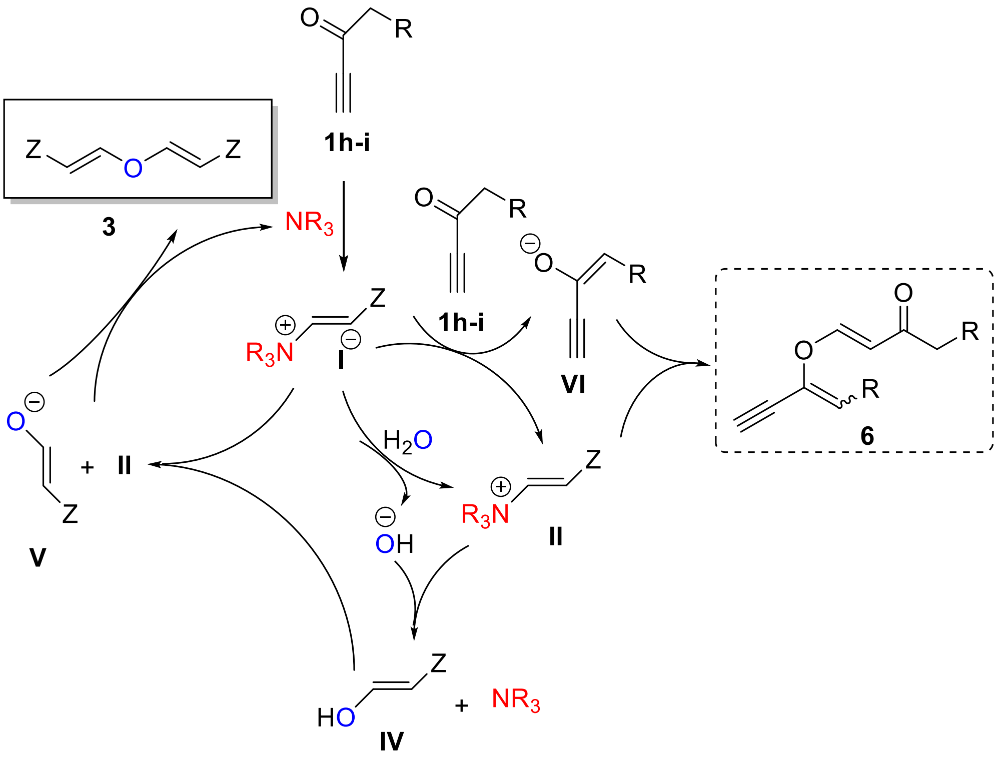

Scheme S2. Mechanistic proposal for the formation of products **3** or **6** when the initial alkyne bears a ketone functionality with α-protons. The acidity of those protons competes with the acidity of water so that intermediate I can be protonated by either water or the starting alkyne **1h-i**, leading to **3** or **6**. Z = $C(O)CH_2R$

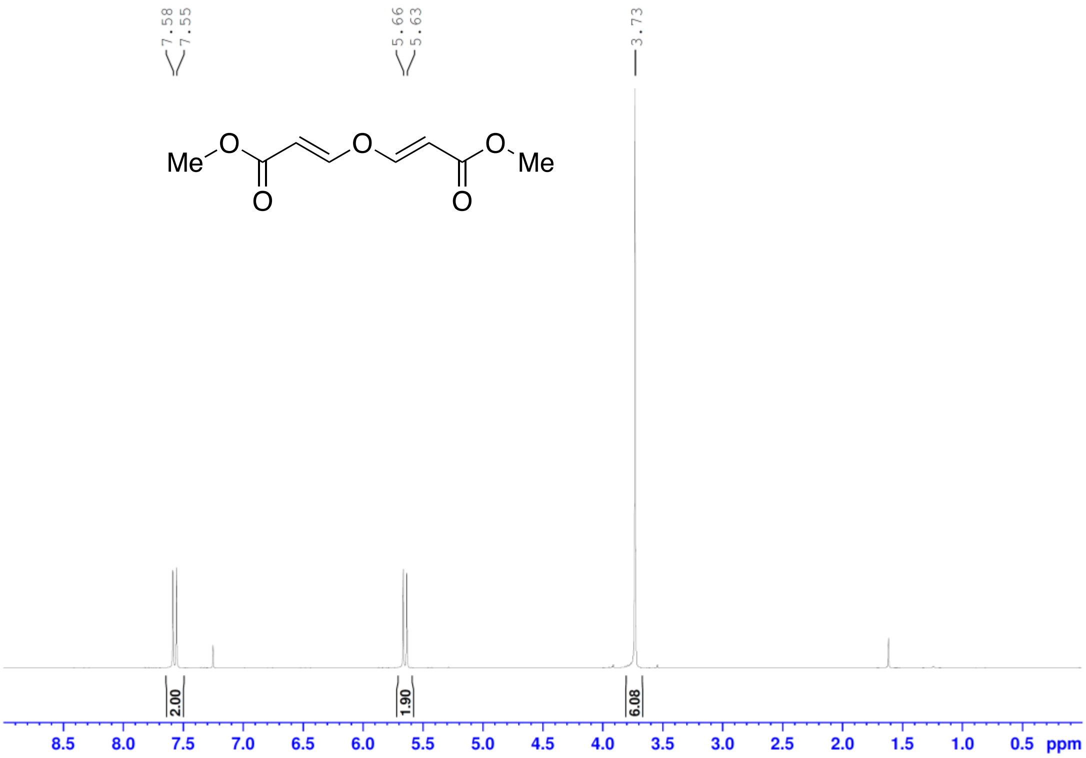

^1H NMR (400 MHz, $CDCl_3$) of compound **3a** (***E*,*E***)

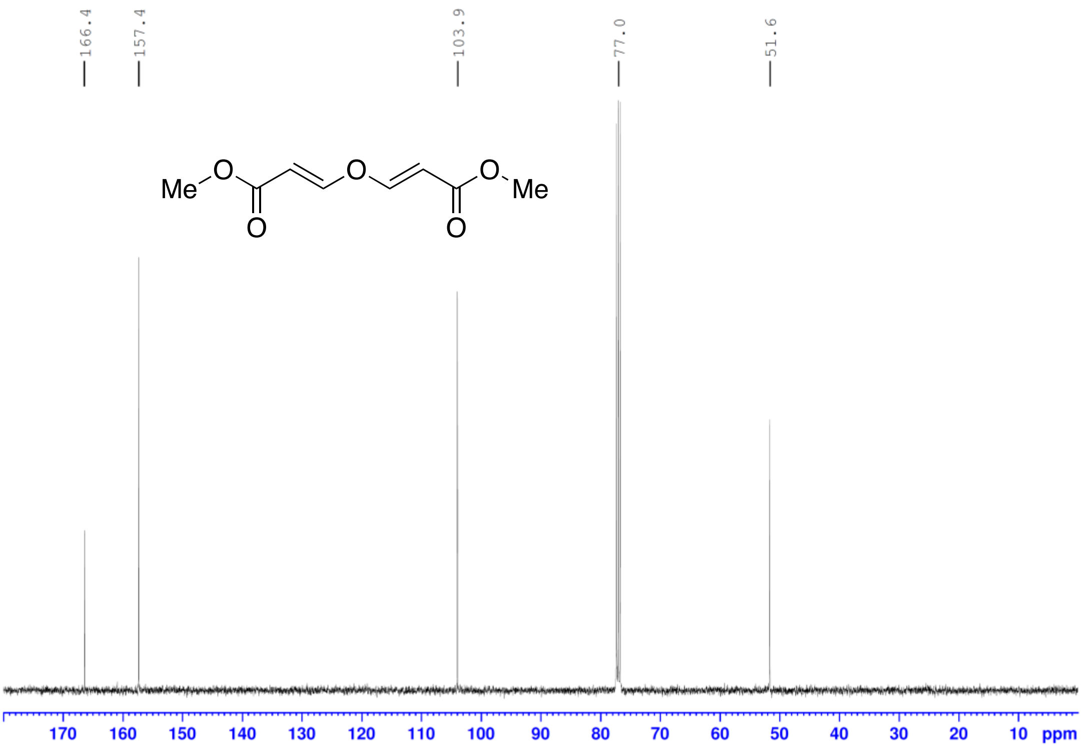

$^{13}C\{^1H\}$ NMR (100 MHz, $CDCl_3$) of compound **3a** (***E*,*E***)

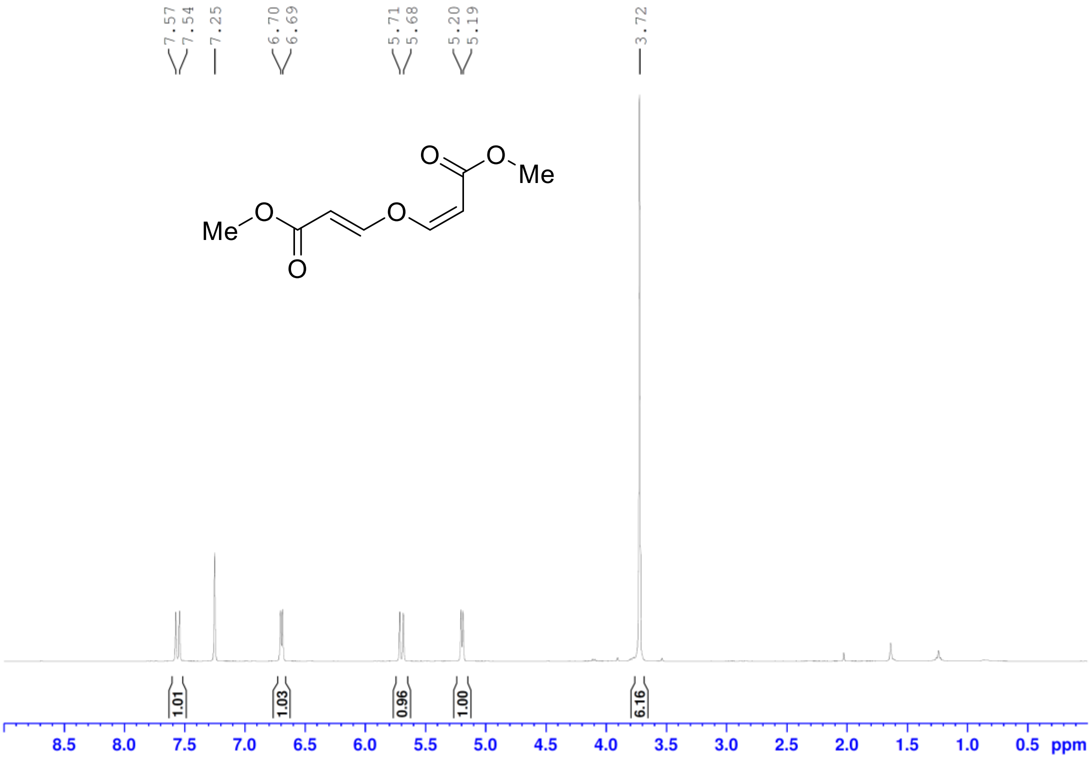

^{1}H NMR (500 MHz, $CDCl_3$) of compound **3a** (***E*,*Z***)

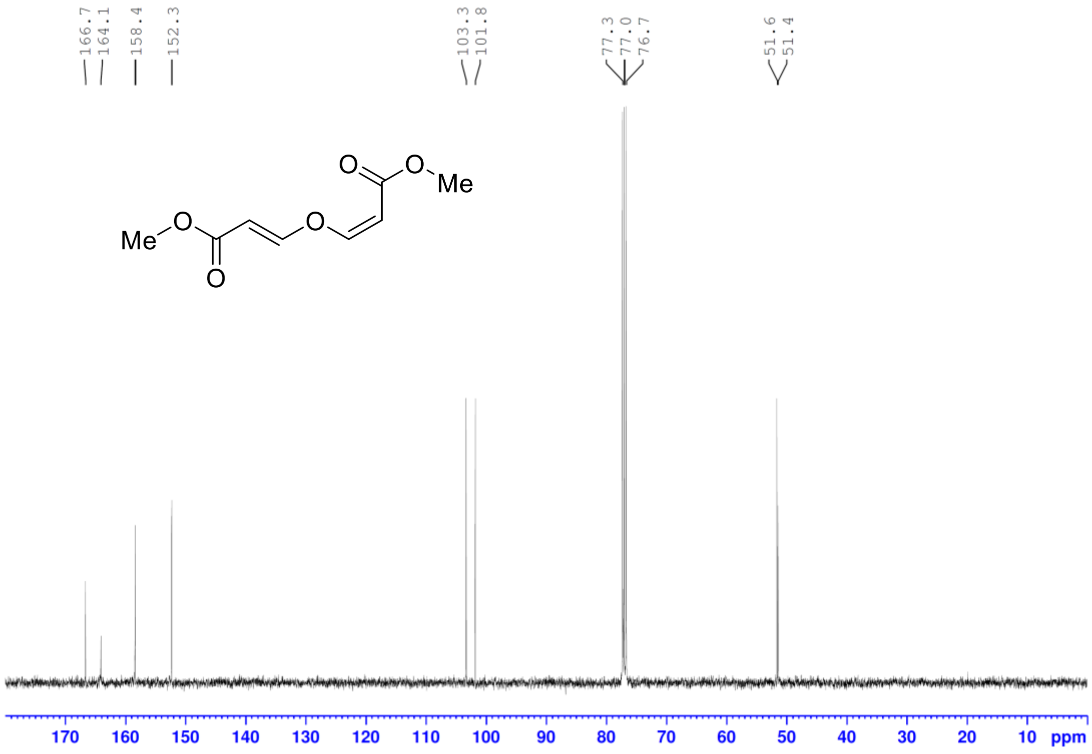

^{13}C{^{1}H} NMR (126 MHz, $CDCl_3$) of compound **3a** (***E*,*Z***)

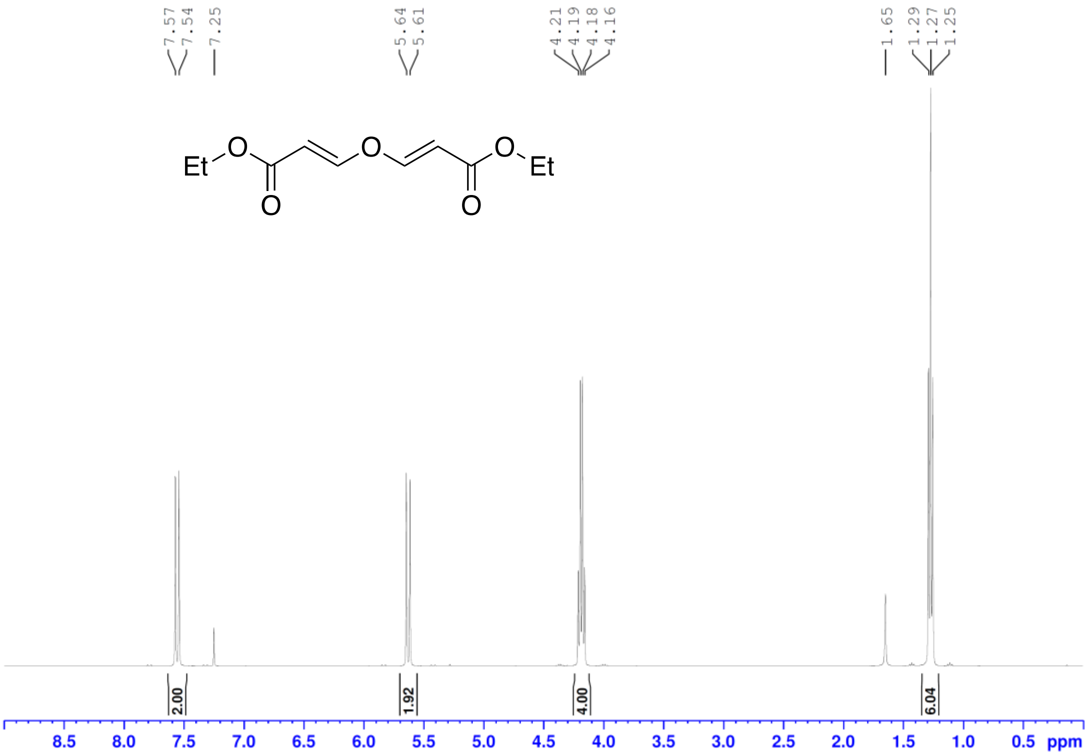

^{1}H NMR (400 MHz, $CDCl_3$) of compound **3b** (***E*,*E***)

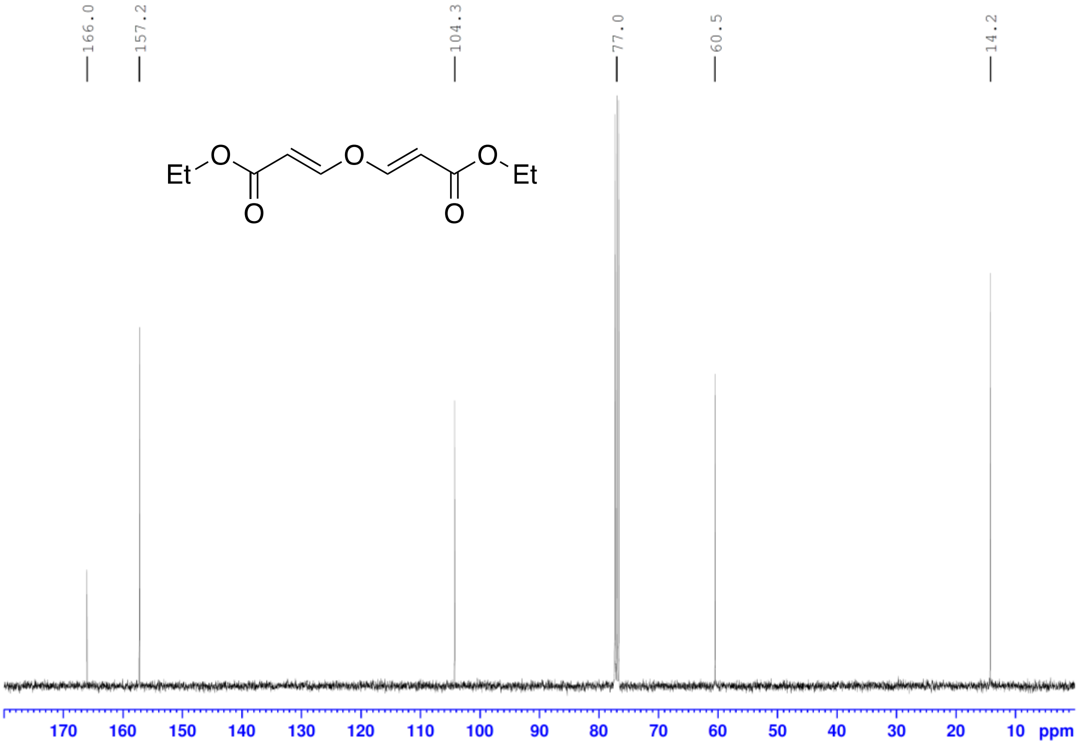

^{13}C{^{1}H} NMR (100 MHz, $CDCl_3$) of compound **3b** (***E*,*E***)

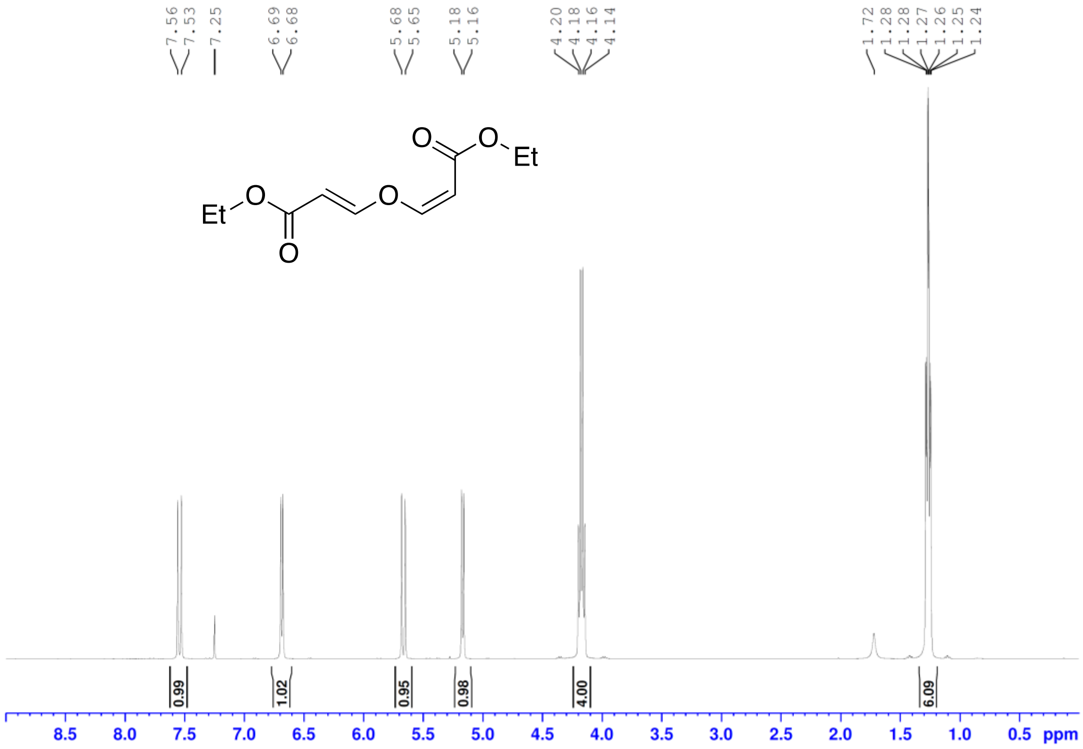

^{1}H NMR (400 MHz, $CDCl_3$) of compound **3b** **(*E*,*Z*)**

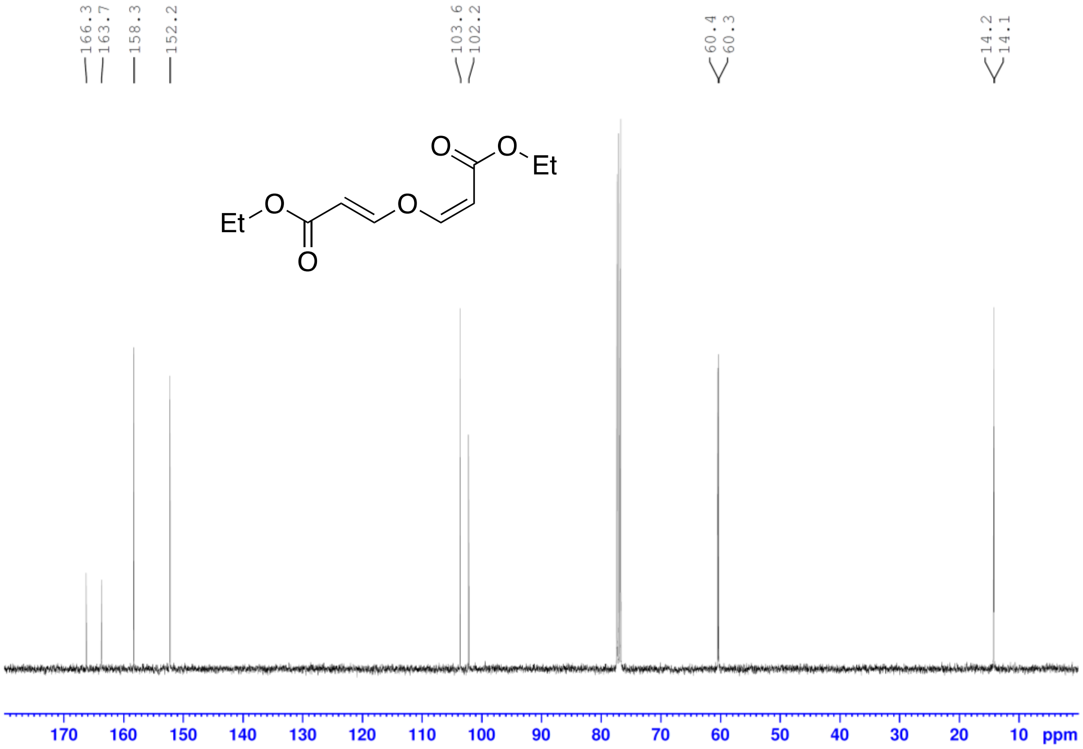

$^{13}C\{^{1}H\}$ NMR (100 MHz, $CDCl_3$) of compound **3b** **(*E*,*Z*)**

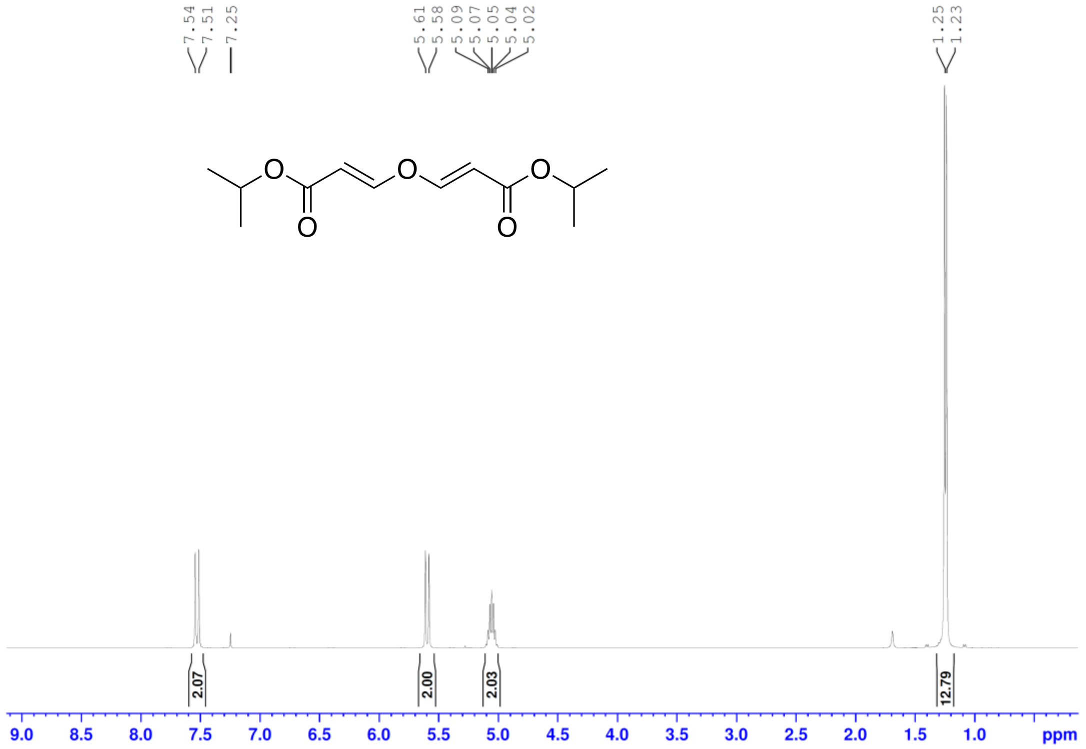

^{1}H NMR (400 MHz, $CDCl_3$) of compound **3c (*E*,*E*)**

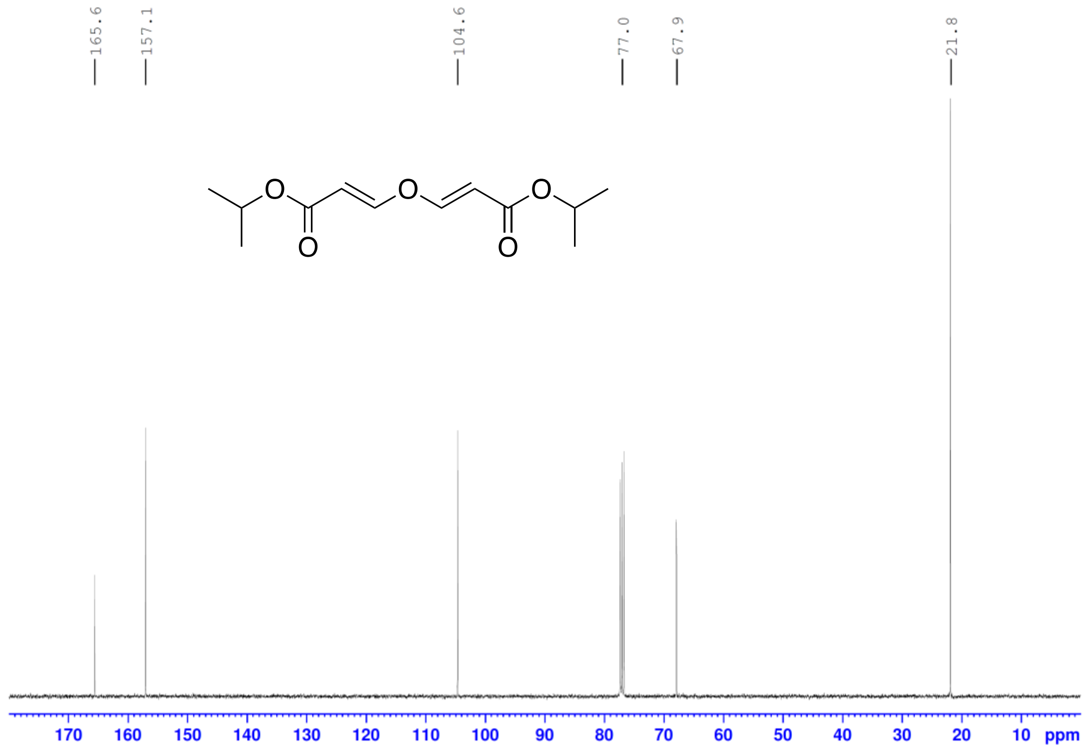

$^{13}C\{^{1}H\}$ NMR (100 MHz, $CDCl_3$) of compound **3c (*E*,*E*)**

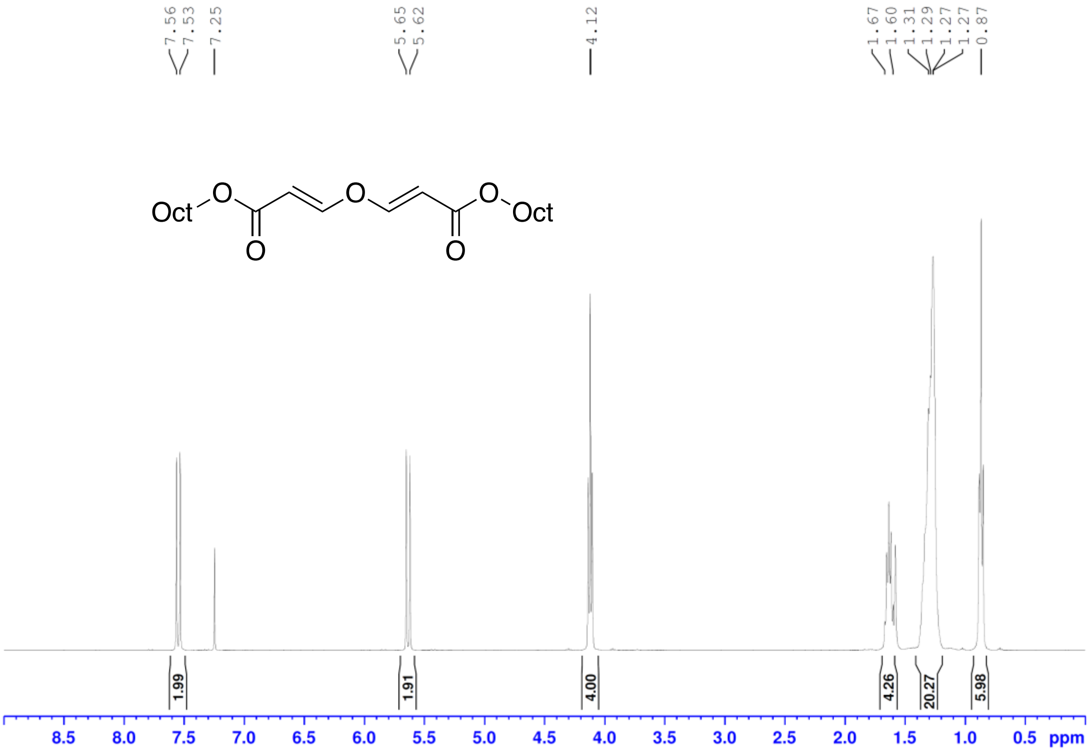

^{1}H NMR (400 MHz, $CDCl_3$) of compound **3d** (***E*,*E***)

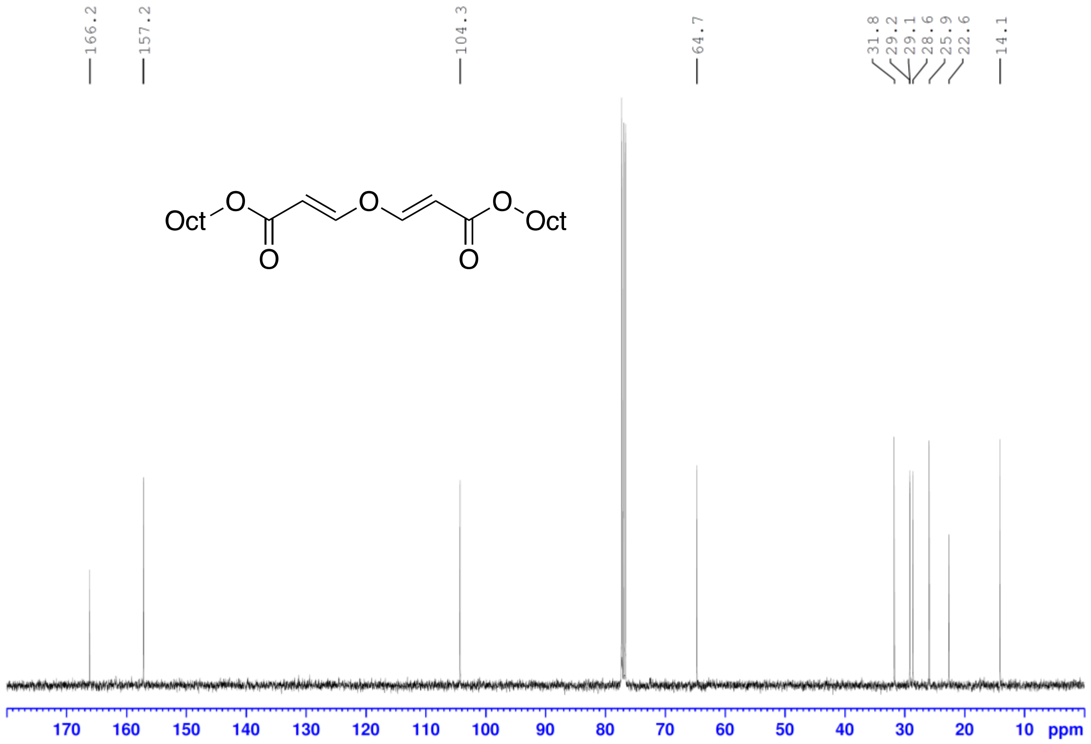

$^{13}C\{^{1}H\}$ NMR (100 MHz, $CDCl_3$) of compound **3d** (***E*,*E***)

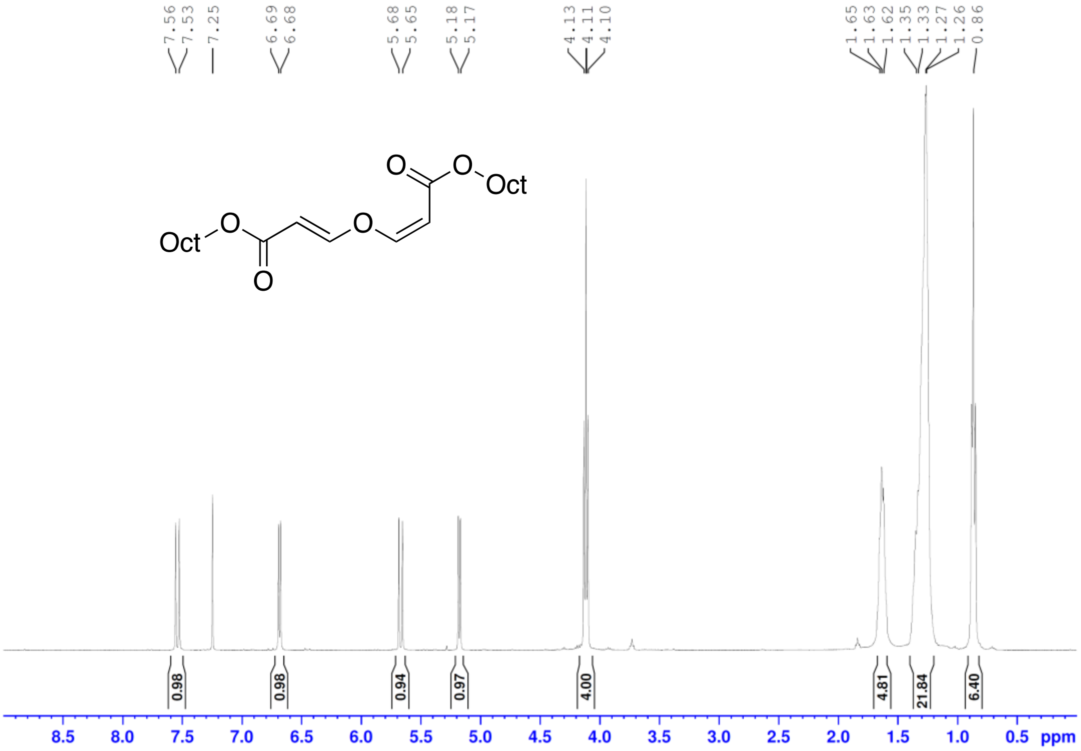

^{1}H NMR (400 MHz, $CDCl_3$) of compound **3d** (***E*,*Z***)

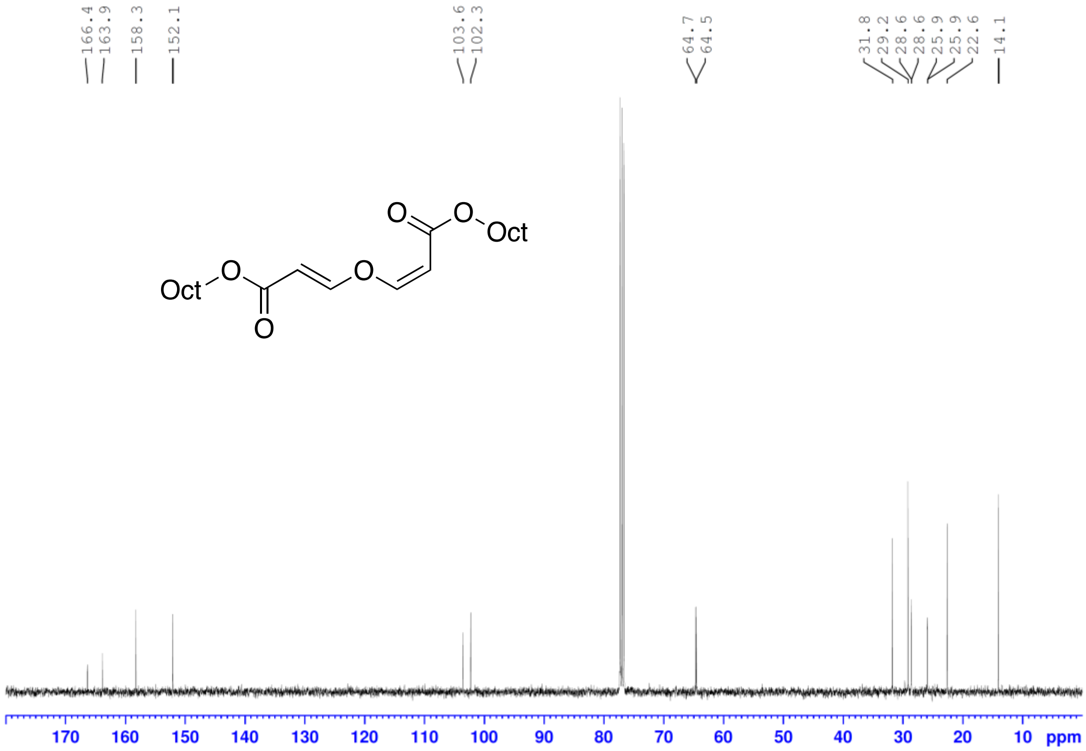

^{13}C{^{1}H} NMR (100 MHz, $CDCl_3$) of compound **3d** (***E*,*Z***)

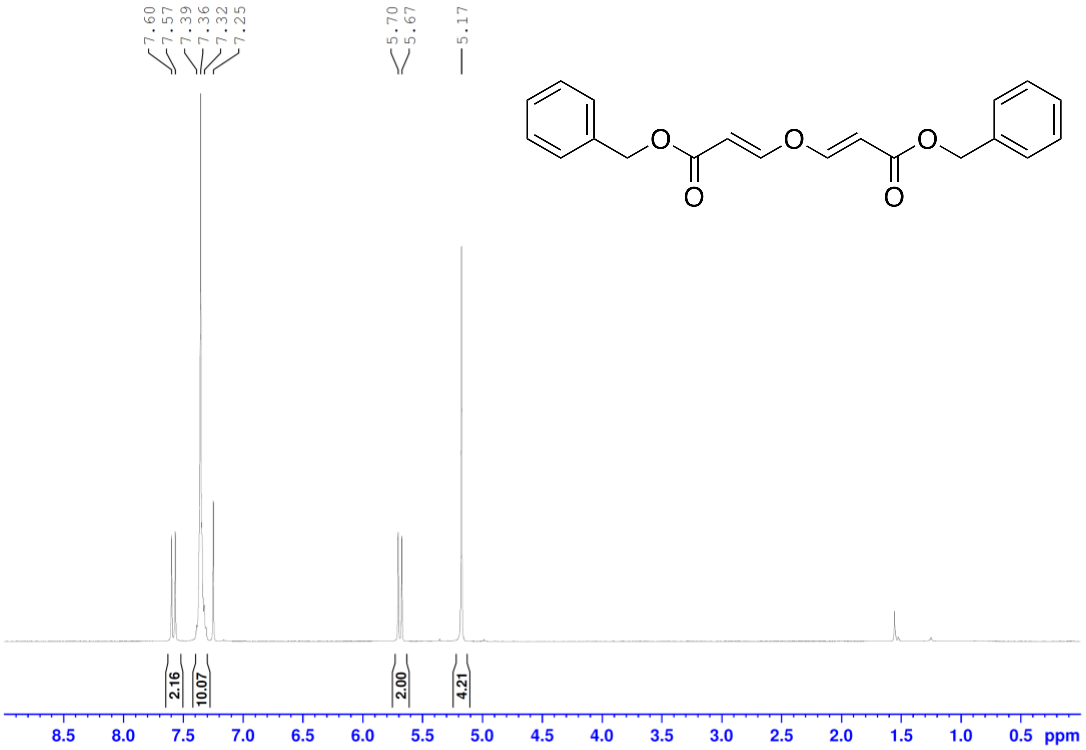

^{1}H NMR (400 MHz, $CDCl_3$) of compound **3e** (***E*,*E***)

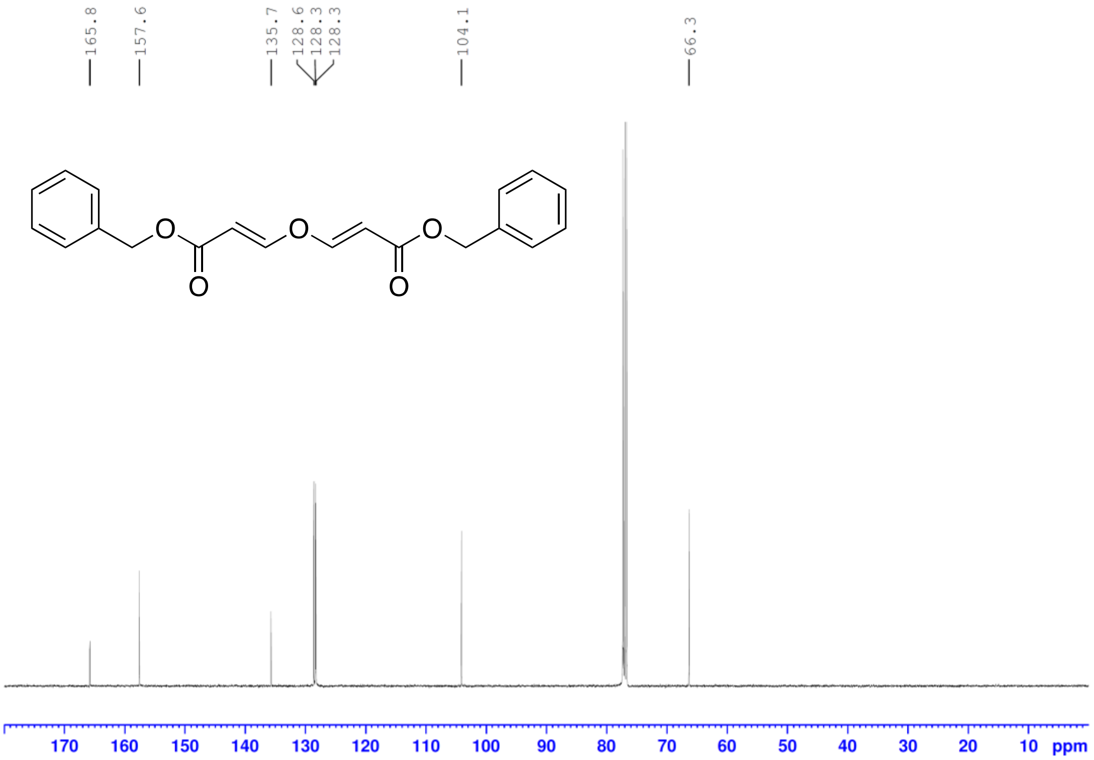

$^{13}C\{^{1}H\}$ NMR (100 MHz, $CDCl_3$) of compound **3e** (***E*,*E***)

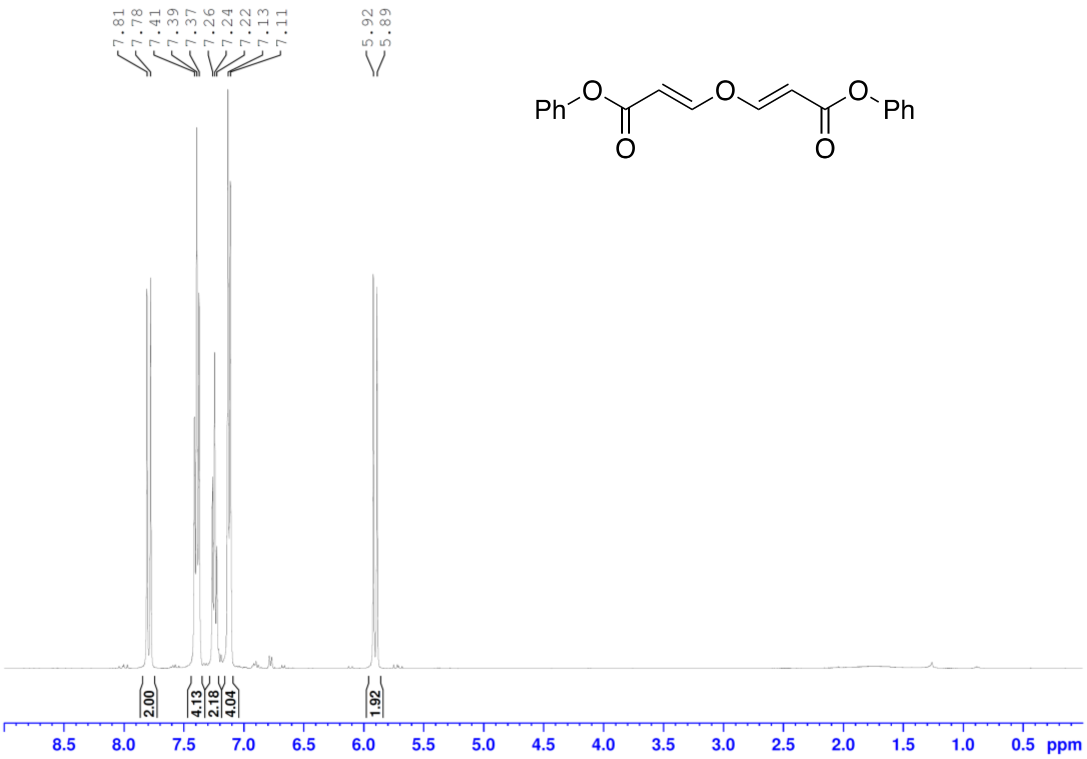

^{1}H NMR (400 MHz, $CDCl_3$) of compound **3f** (***E*,*E***)

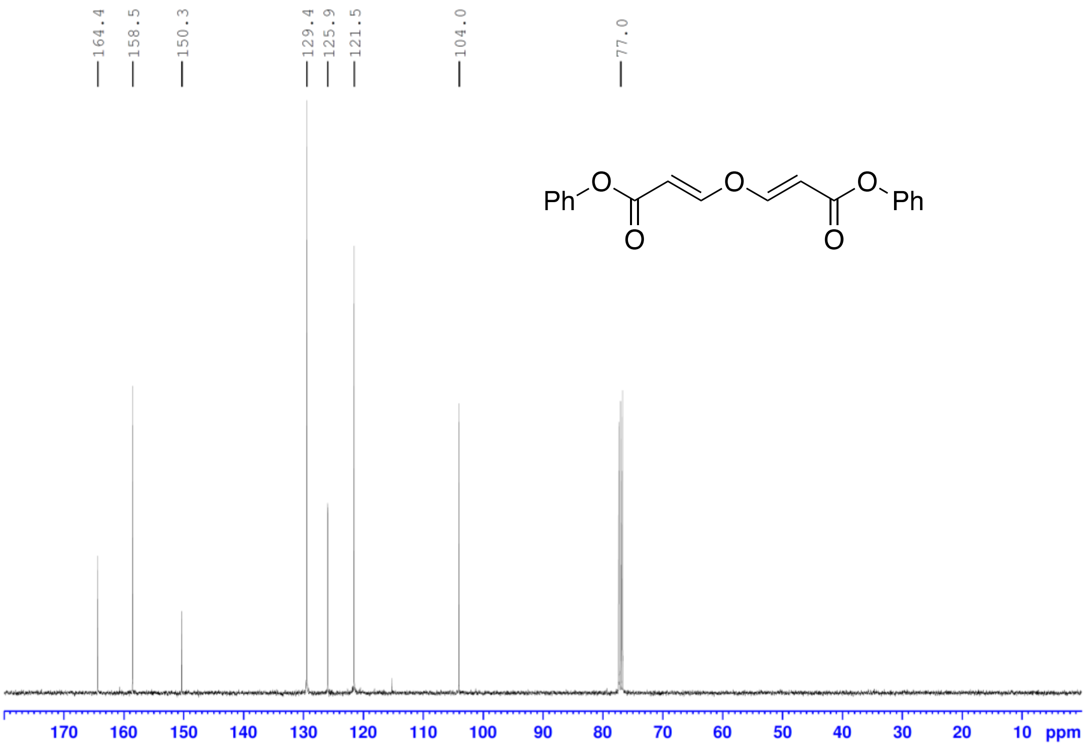

$^{13}C\{^{1}H\}$ NMR (100 MHz, $CDCl_3$) of compound **3f** (***E*,*E***)

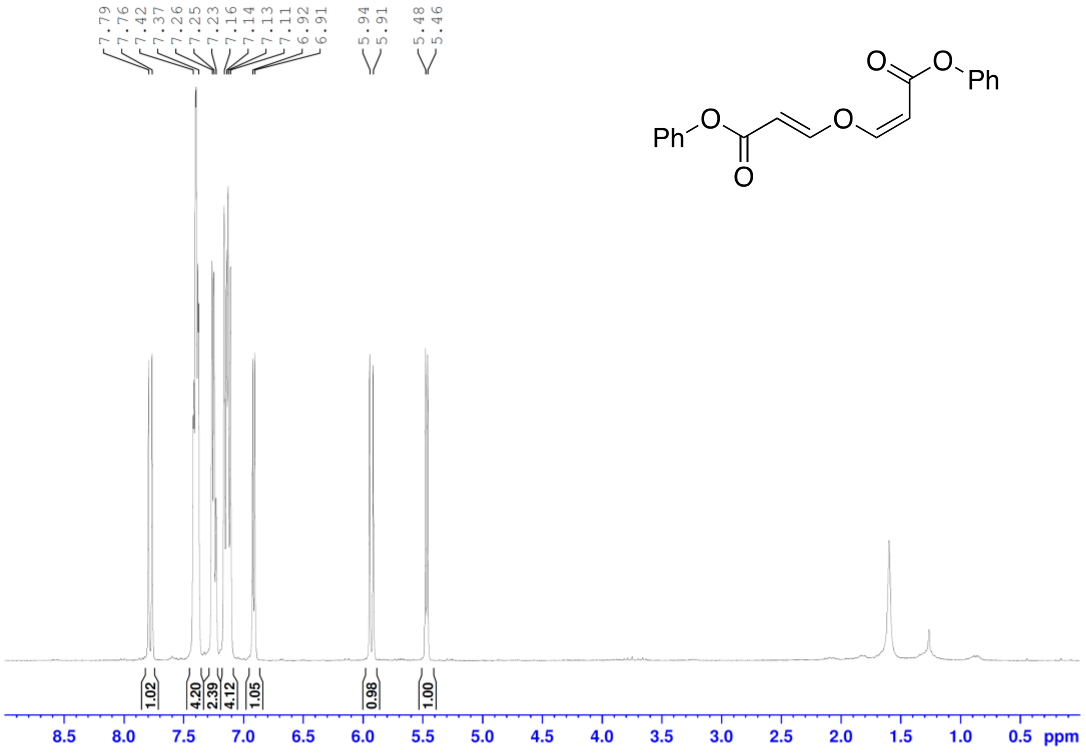

^{1}H NMR (400 MHz, $CDCl_3$) of compound **3f** (***E*,*Z***)

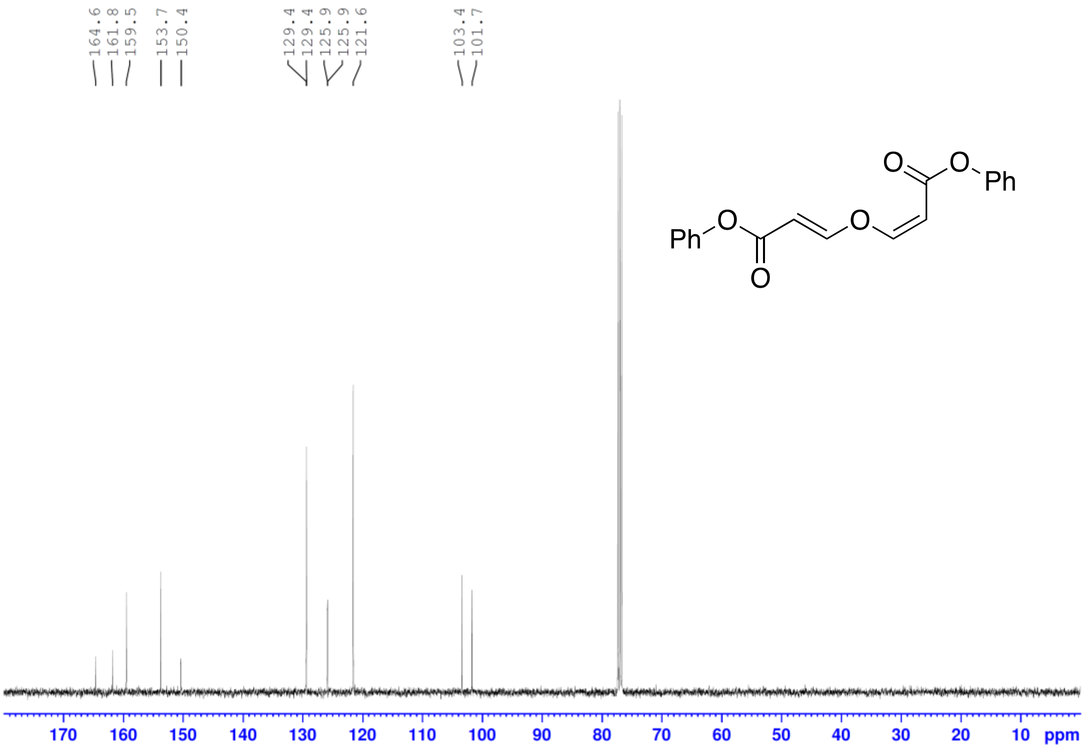

$^{13}C\{^{1}H\}$ NMR (100 MHz, $CDCl_3$) of compound **3f** (***E*,*Z***)

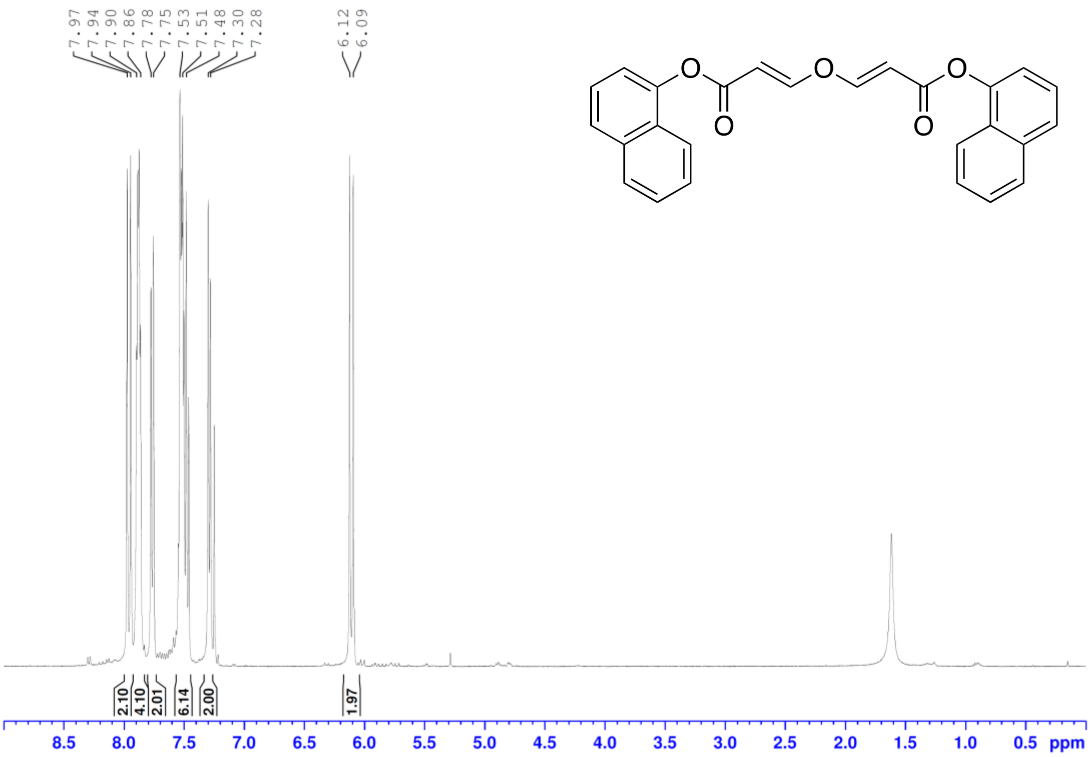

^{1}H NMR (400 MHz, $CDCl_3$) of compound **3g** **(*E*,*E*)**

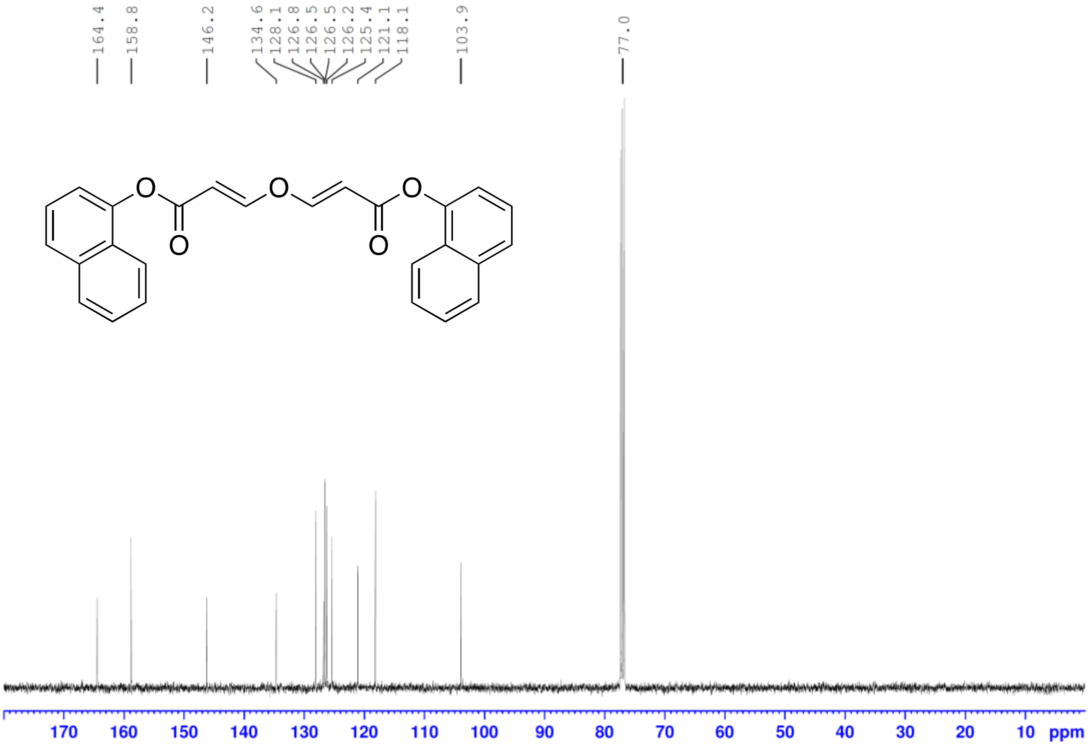

^{13}C{^{1}H} NMR (100 MHz, $CDCl_3$) of compound **3g** **(*E*,*E*)**

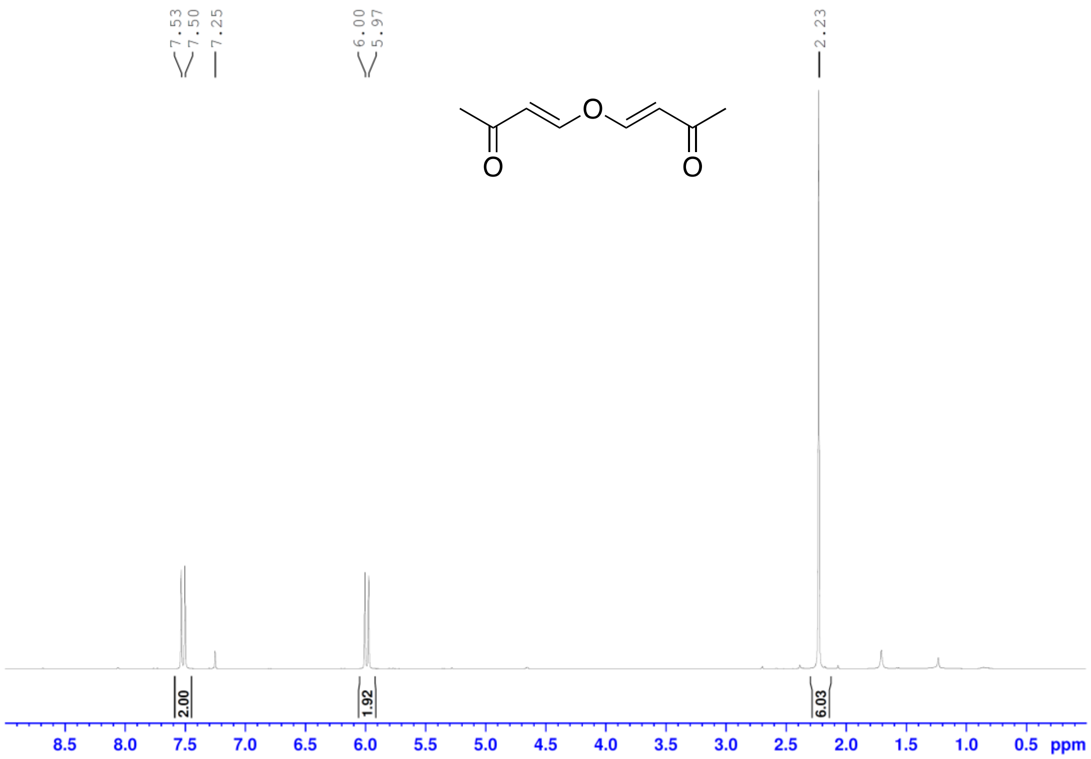

^{1}H NMR (400 MHz, $CDCl_3$) of compound **3h (*E*,*E*)**

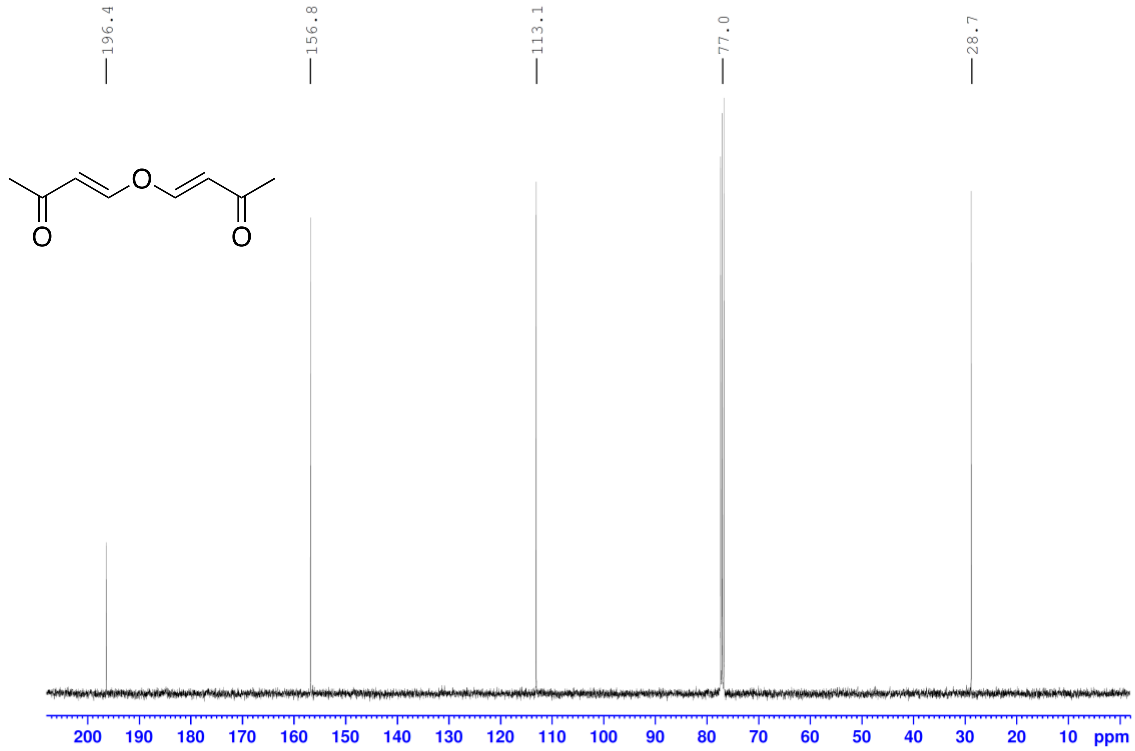

$^{13}C\{^{1}H\}$ NMR (100 MHz, $CDCl_3$) of compound **3h (*E*,*E*)**

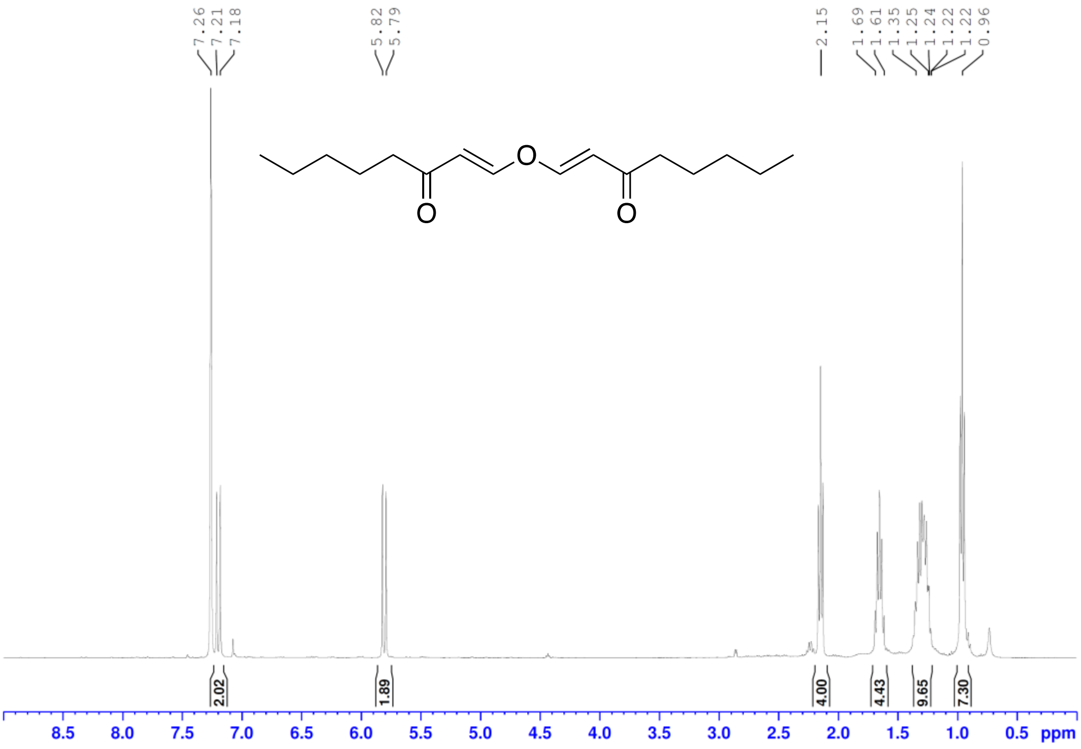

^{1}H NMR (400 MHz, $CDCl_3$) of compound **3i** (***E*,*E***)

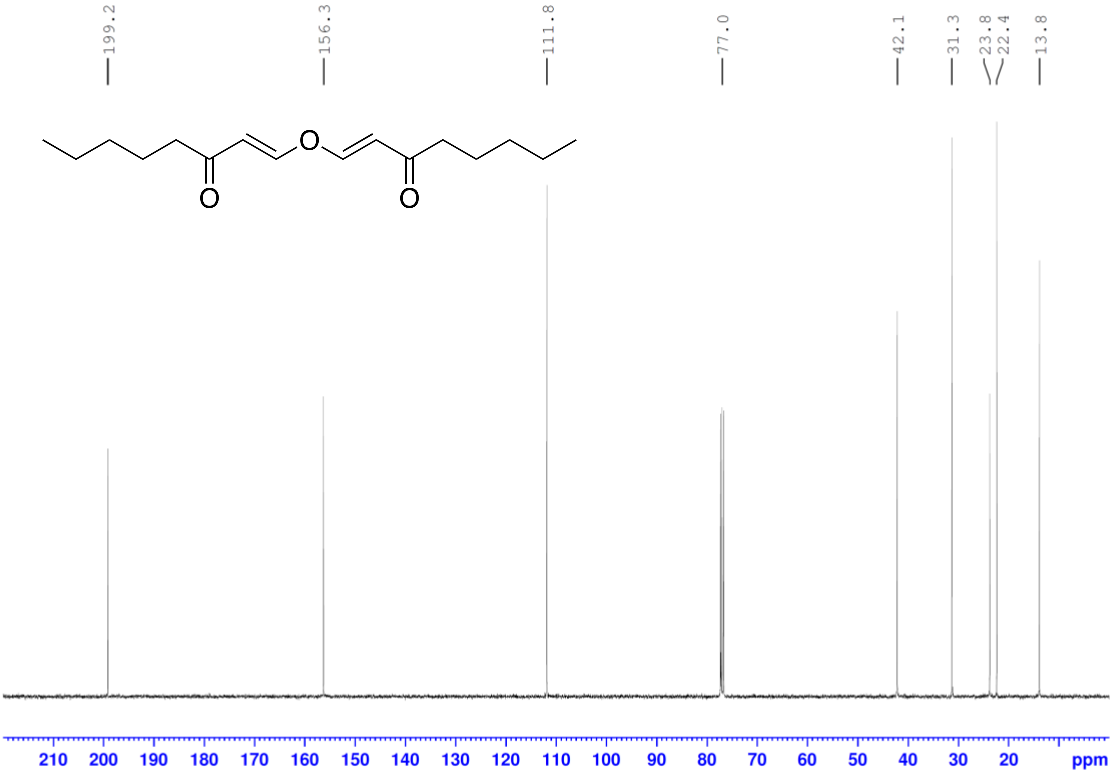

^{13}C{^{1}H} NMR (100 MHz, $CDCl_3$) of compound **3i** (***E*,*E***)

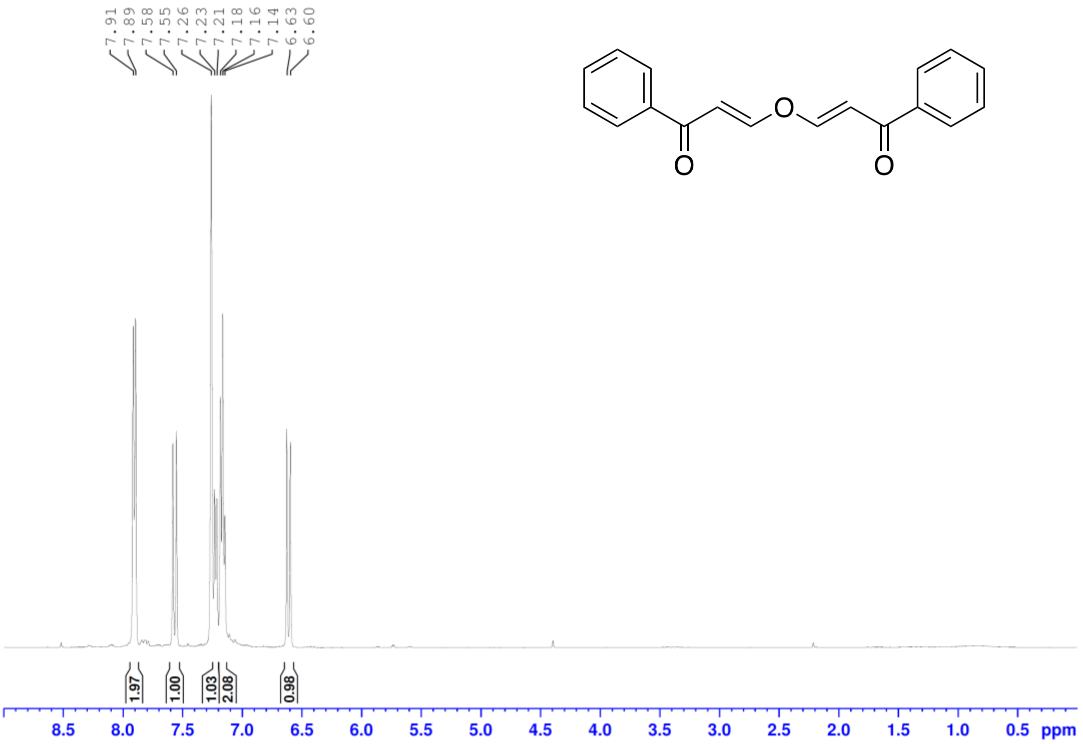

^{1}H NMR (400 MHz, C_6D_6) of compound **3j** (***E*,*E***)

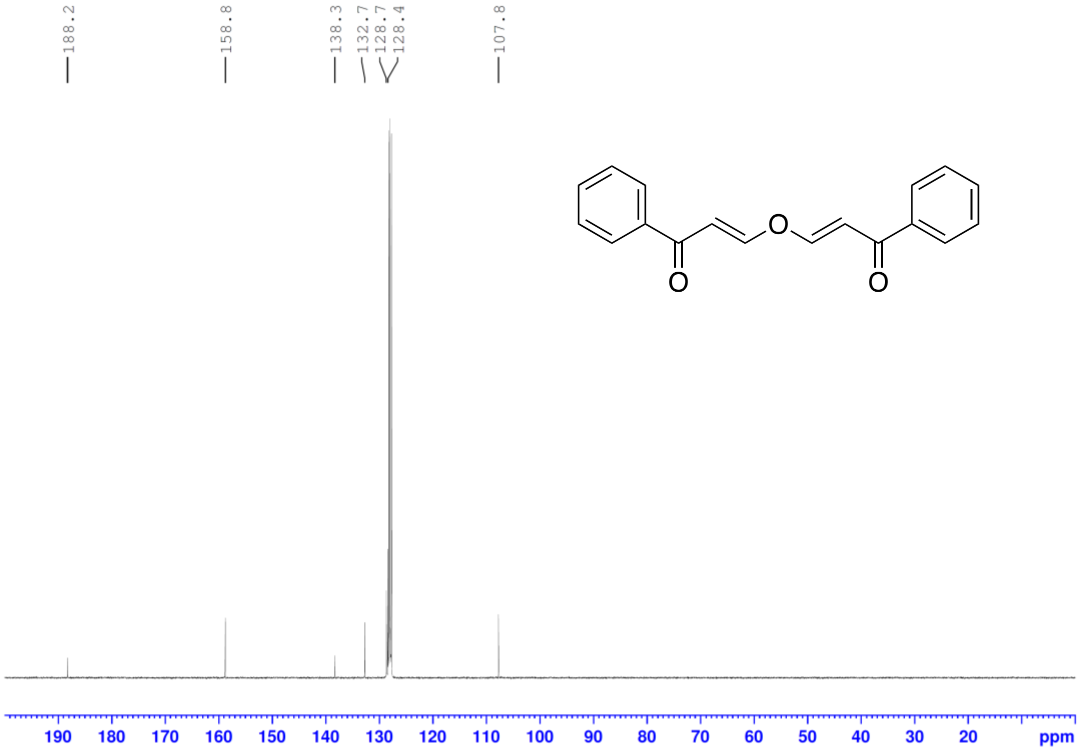

$^{13}C\{^{1}H\}$ NMR (100 MHz, C_6D_6) of compound **3j** (***E*,*E***)

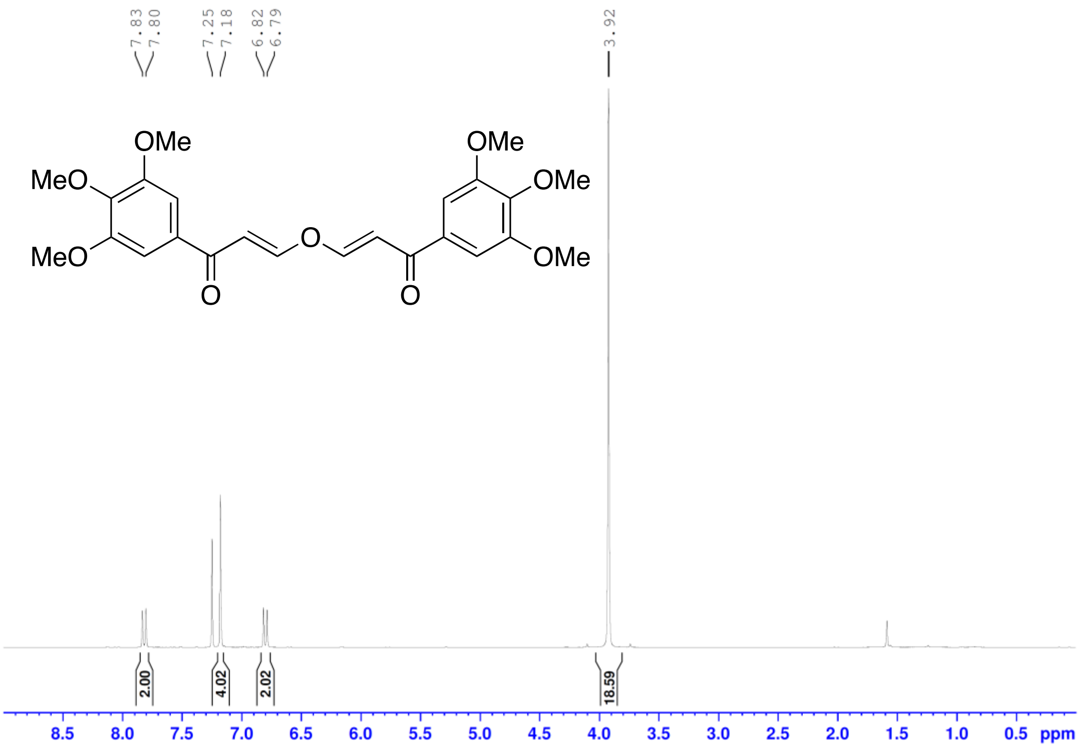

^{1}H NMR (400 MHz, $CDCl_3$) of compound **3k** (***E*,*E***)

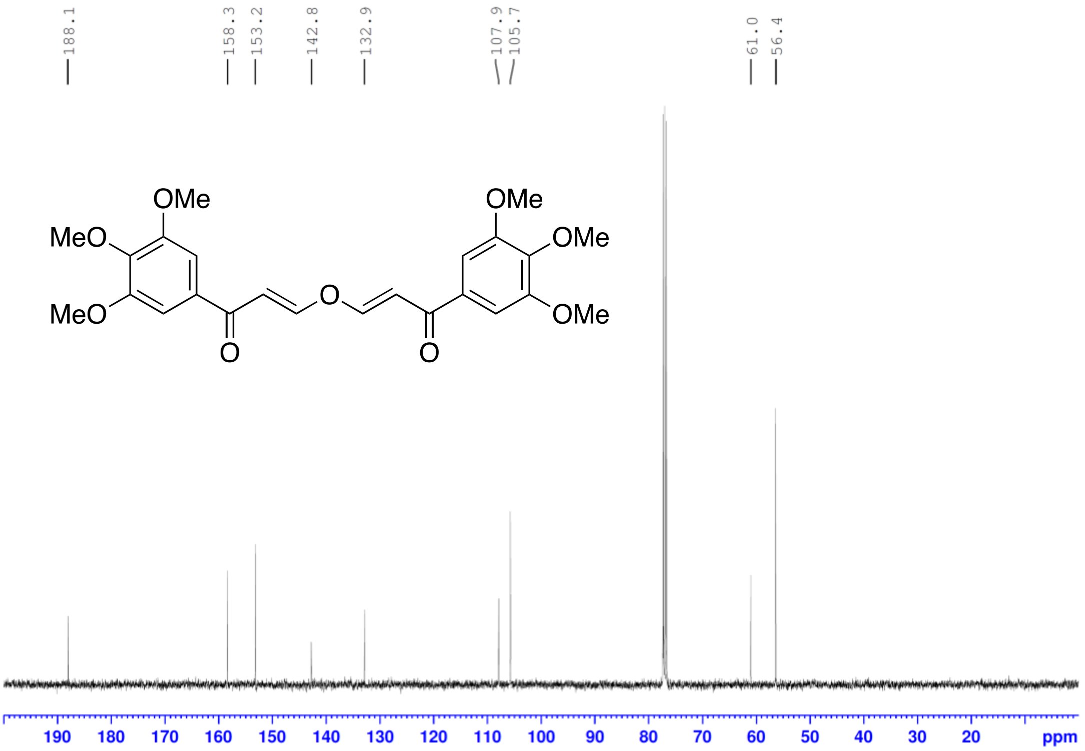

$^{13}C\{^{1}H\}$ NMR (100 MHz, $CDCl_3$) of compound **3k** (***E*,*E***)

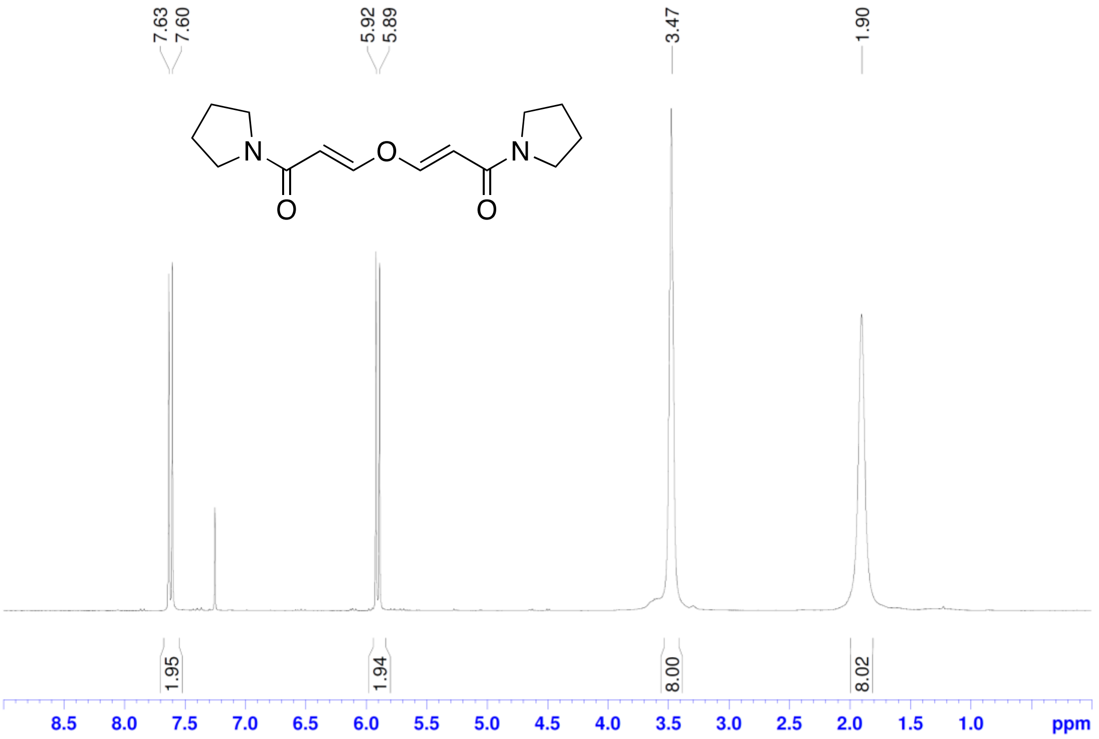

^{1}H NMR (400 MHz, $CDCl_3$) of compound **3l** (***E***,***E***)

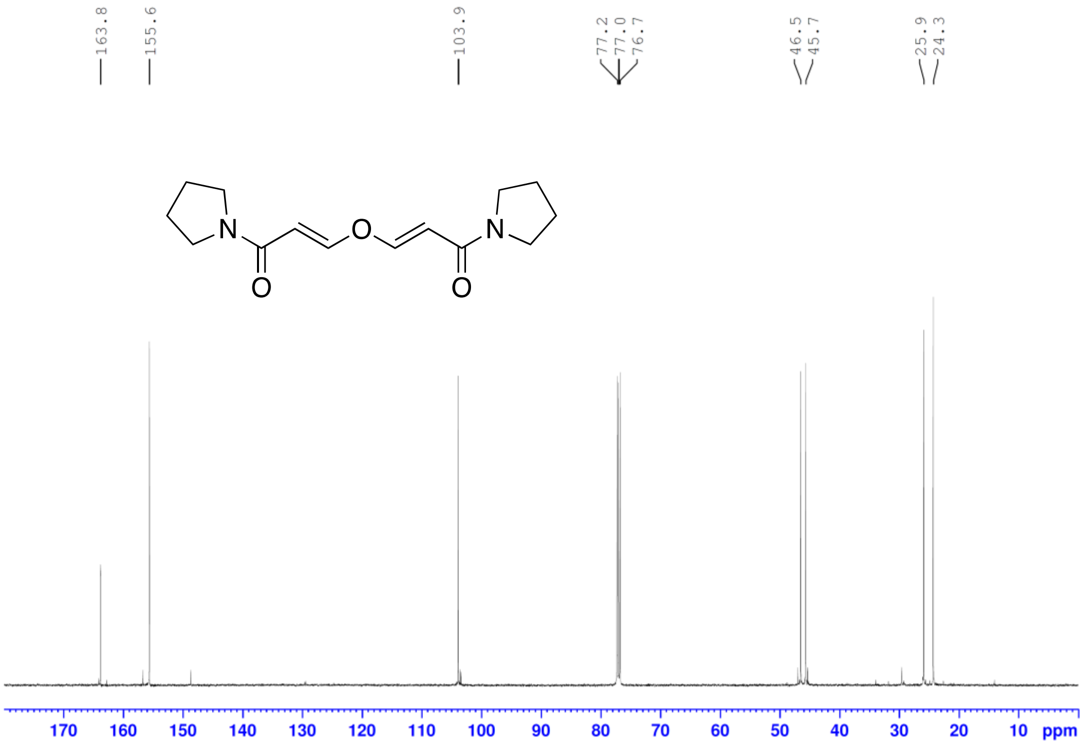

$^{13}C\{^{1}H\}$ NMR (126 MHz, $CDCl_3$) of compound **3l** (***E***,***E***)

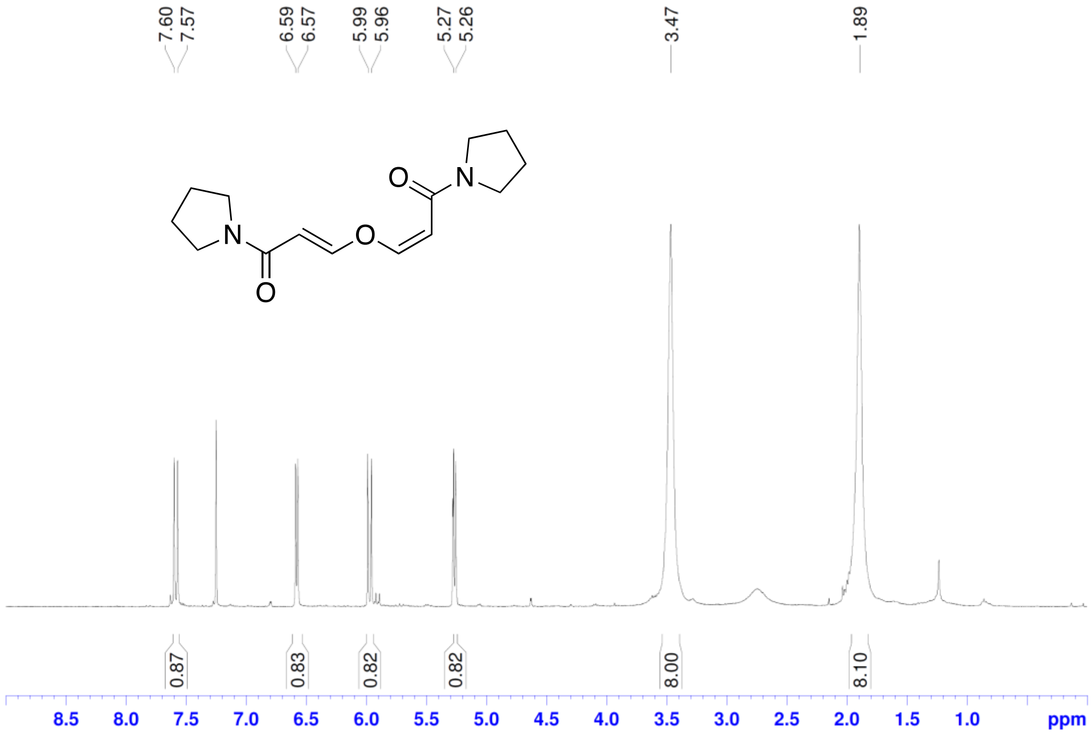

^{1}H NMR (400 MHz, $CDCl_3$) of compound **3l** (***E*,*Z***)

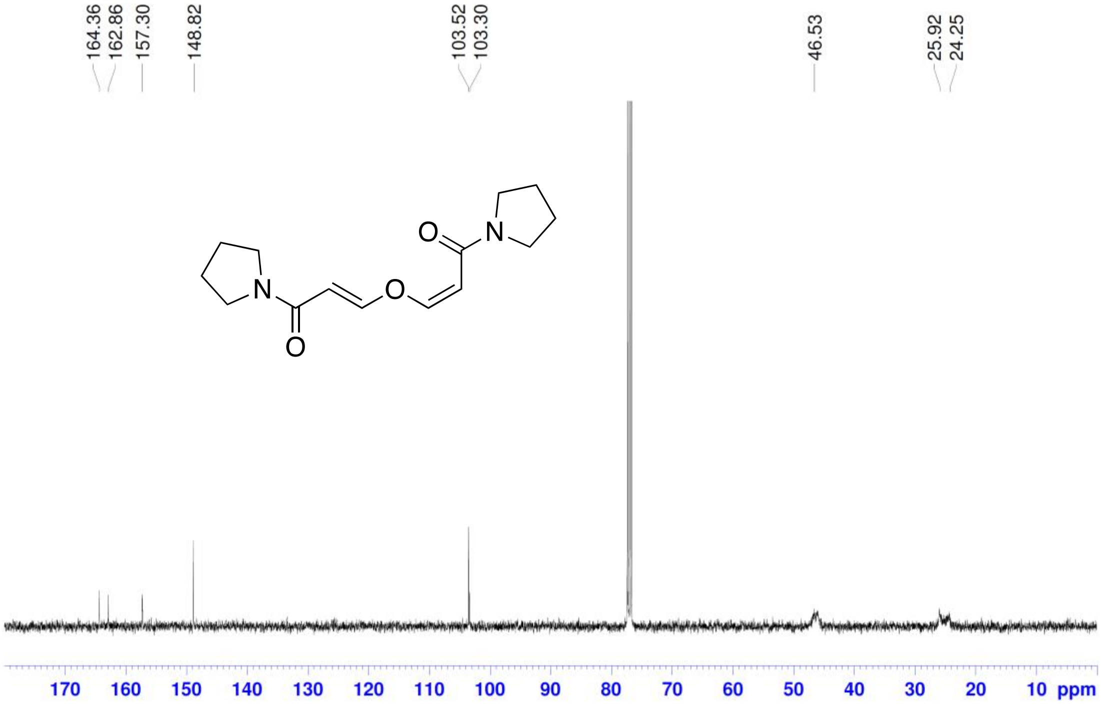

$^{13}C\{^{1}H\}$ NMR (100 MHz, $CDCl_3$) of compound **3l** (***E*,*Z***)

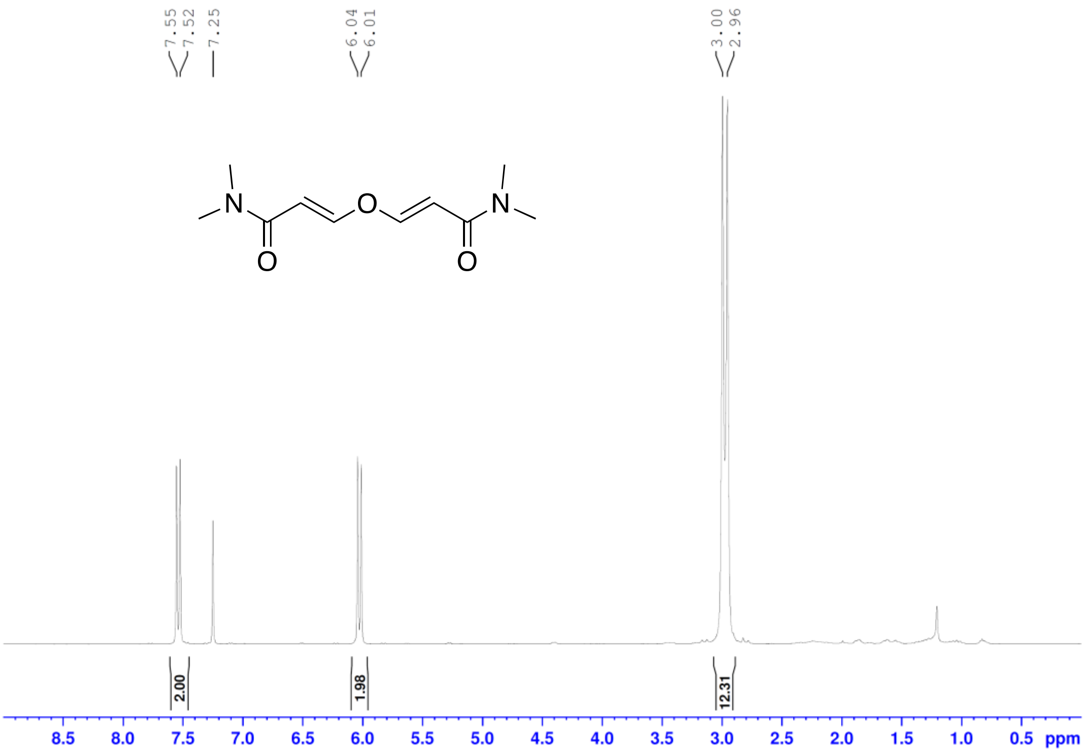

^{1}H NMR (400 MHz, $CDCl_3$) of compound **3m (*E*,*E*)**

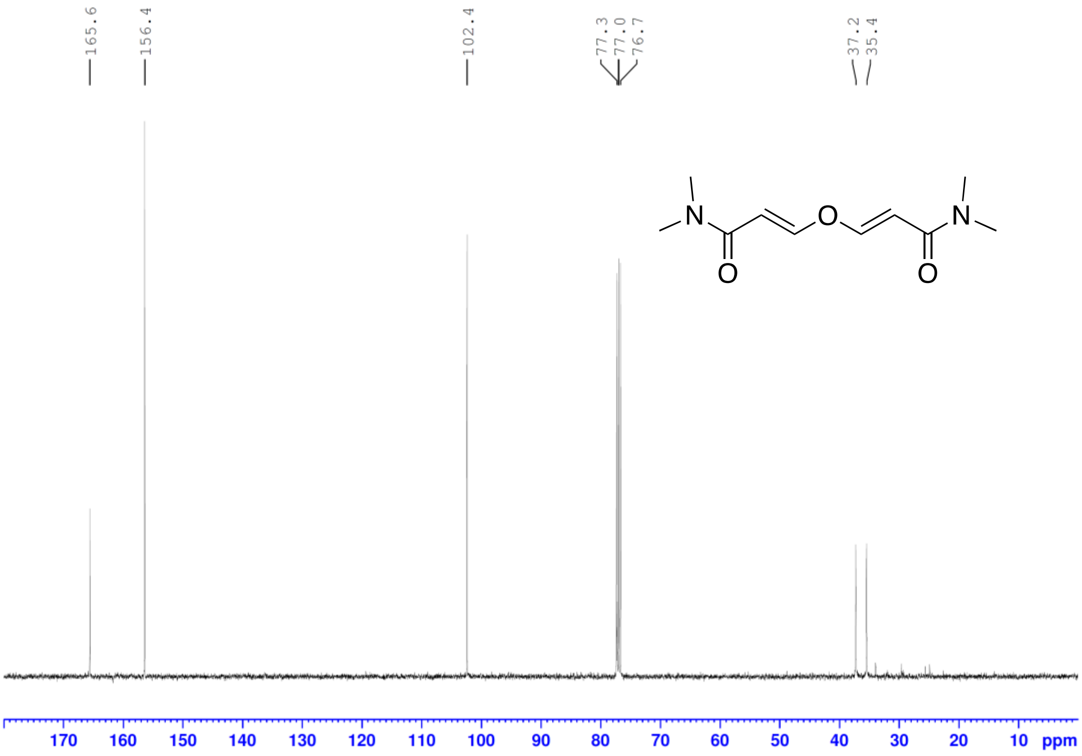

^{13}C{^{1}H} NMR (100 MHz, $CDCl_3$) of compound **3m (*E*,*E*)**

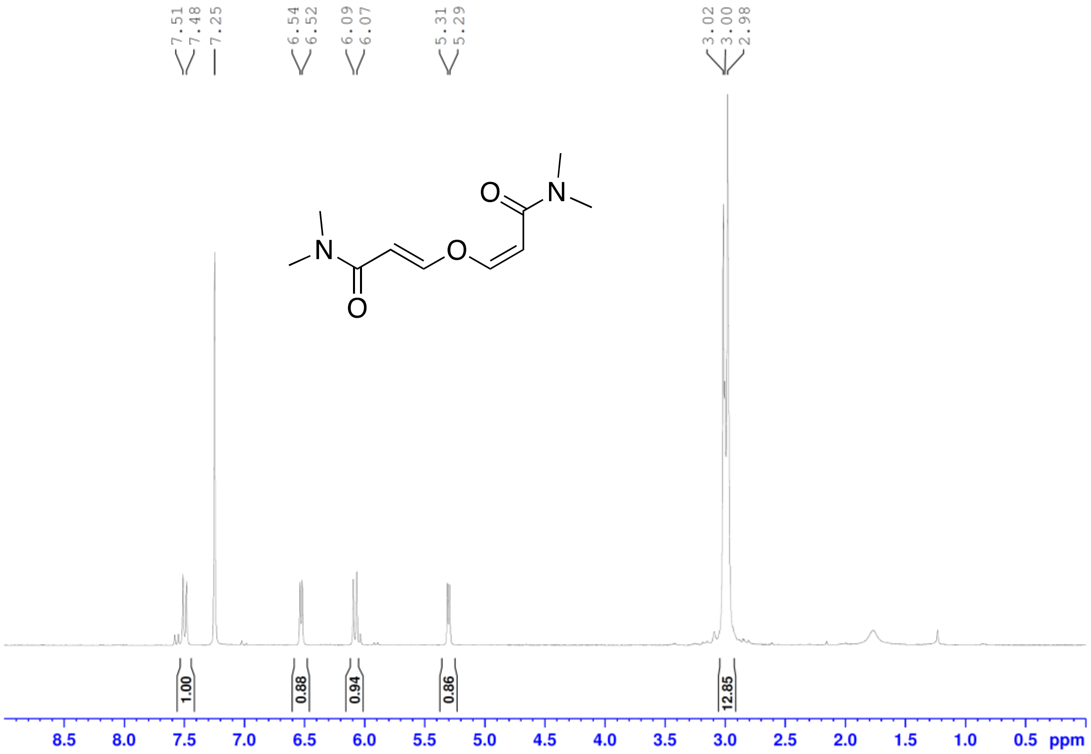

^{1}H NMR (400 MHz, $CDCl_3$) of compound **3m (*E*,*Z*)**. It contains a small amount of the *E*,*E*-isomer

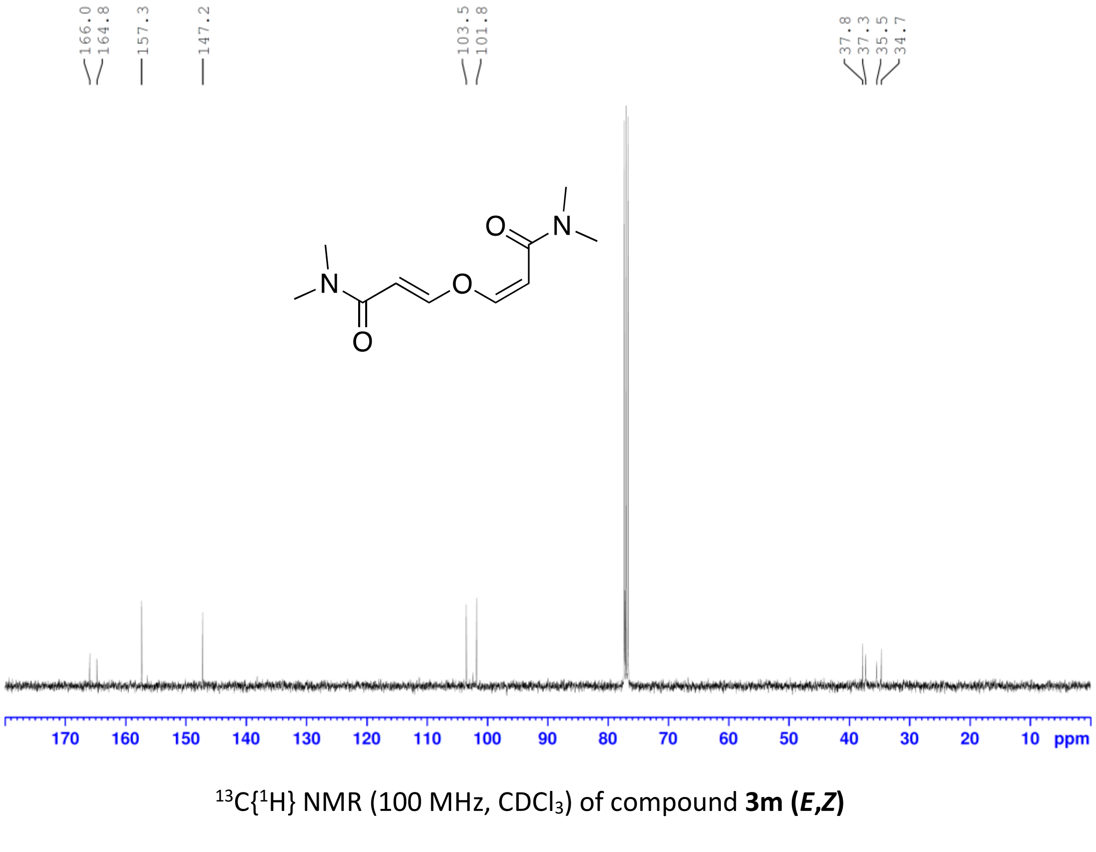

^{13}C{^{1}H} NMR (100 MHz, $CDCl_3$) of compound **3m (*E*,*Z*)**

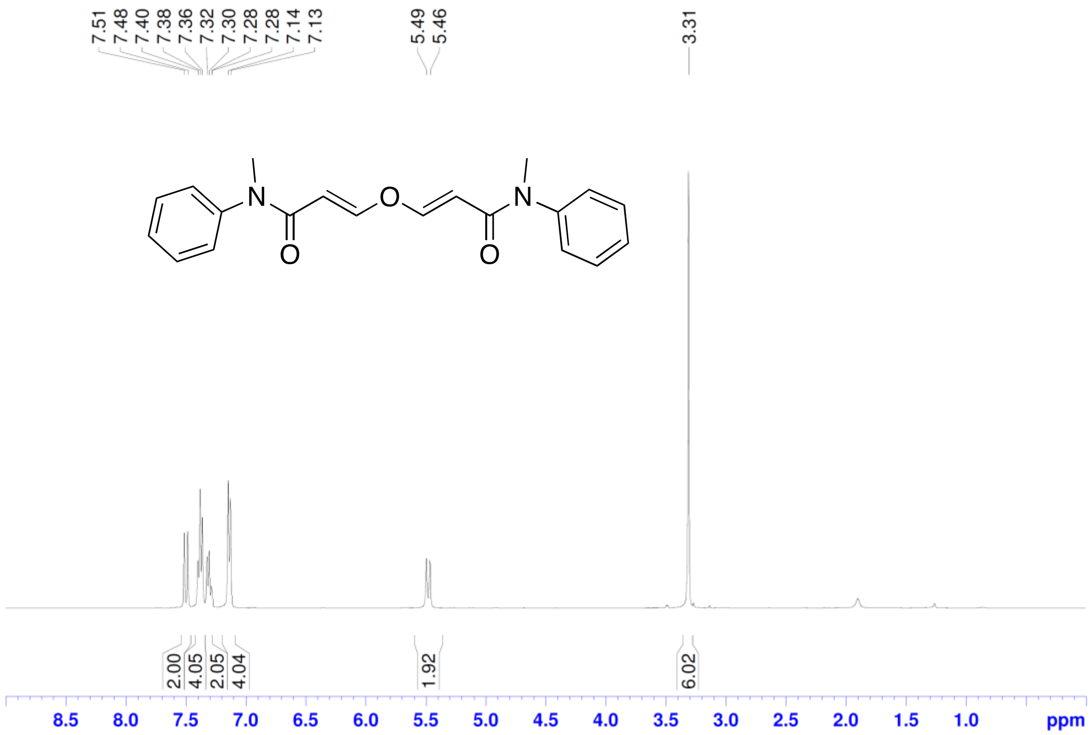

^{1}H NMR (400 MHz, $CDCl_3$) of compound **3n** (***E***,***E***)

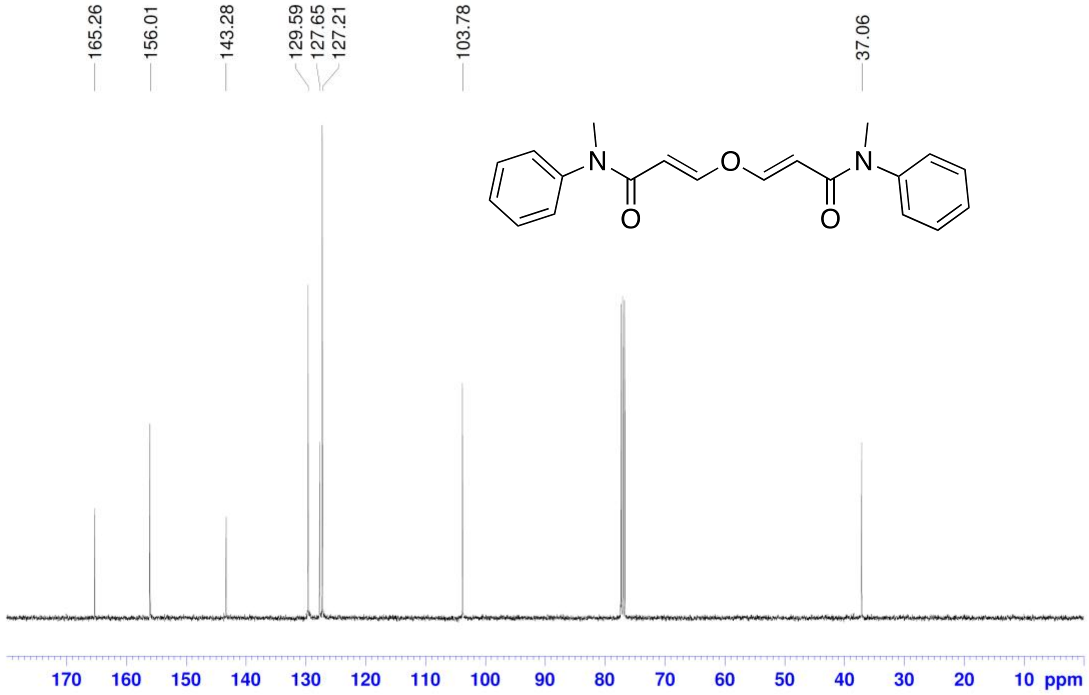

^{13}C{^{1}H} NMR (100 MHz, $CDCl_3$) of compound **3n** (***E***,***E***)

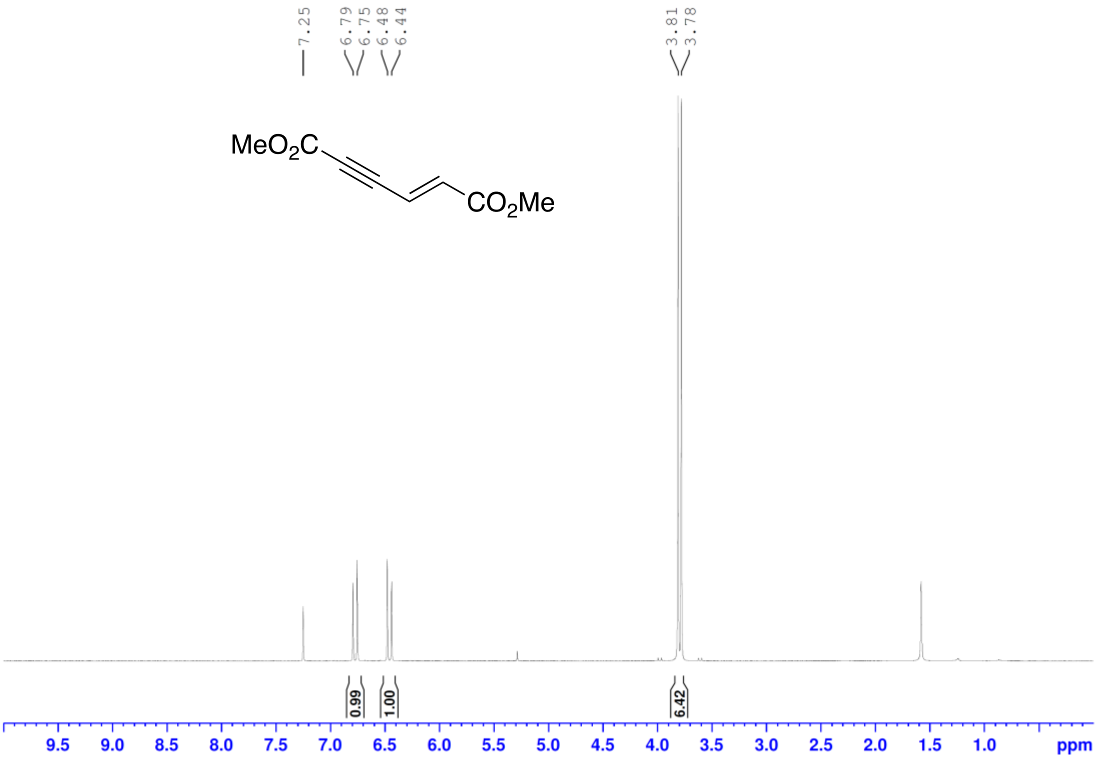

^{1}H NMR (400 MHz, $CDCl_3$) of compound **4a** (***E***)

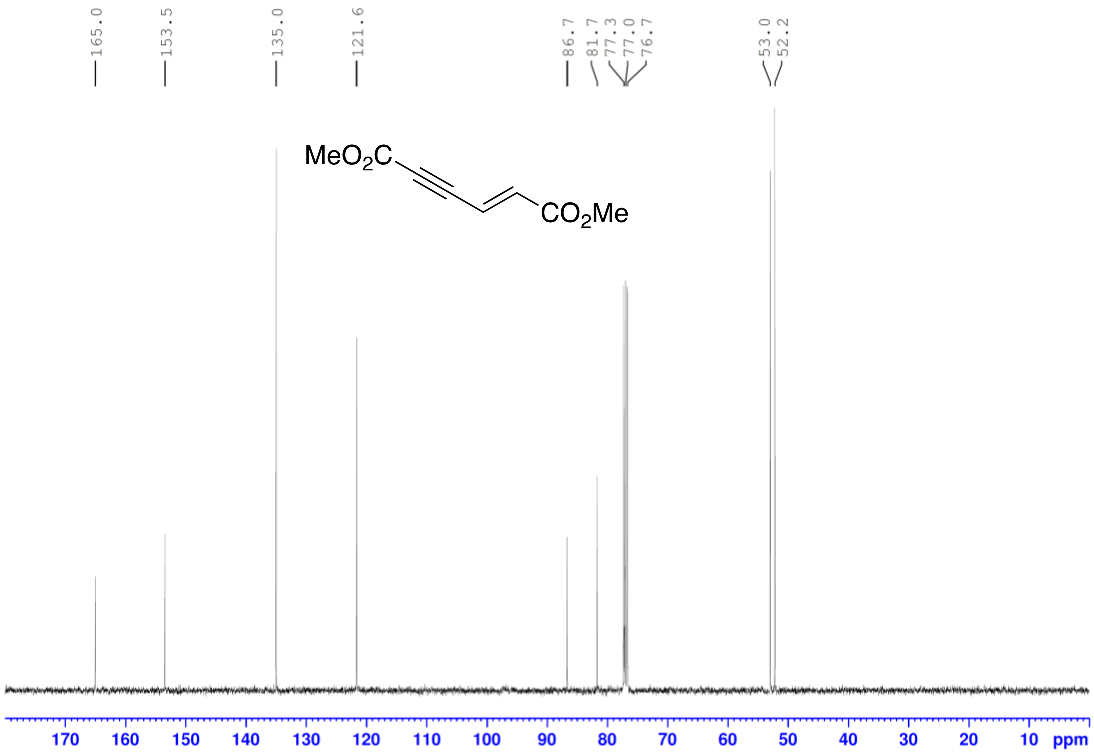

$^{13}C\{^{1}H\}$ NMR (100 MHz, $CDCl_3$) of compound **4a** (***E***)

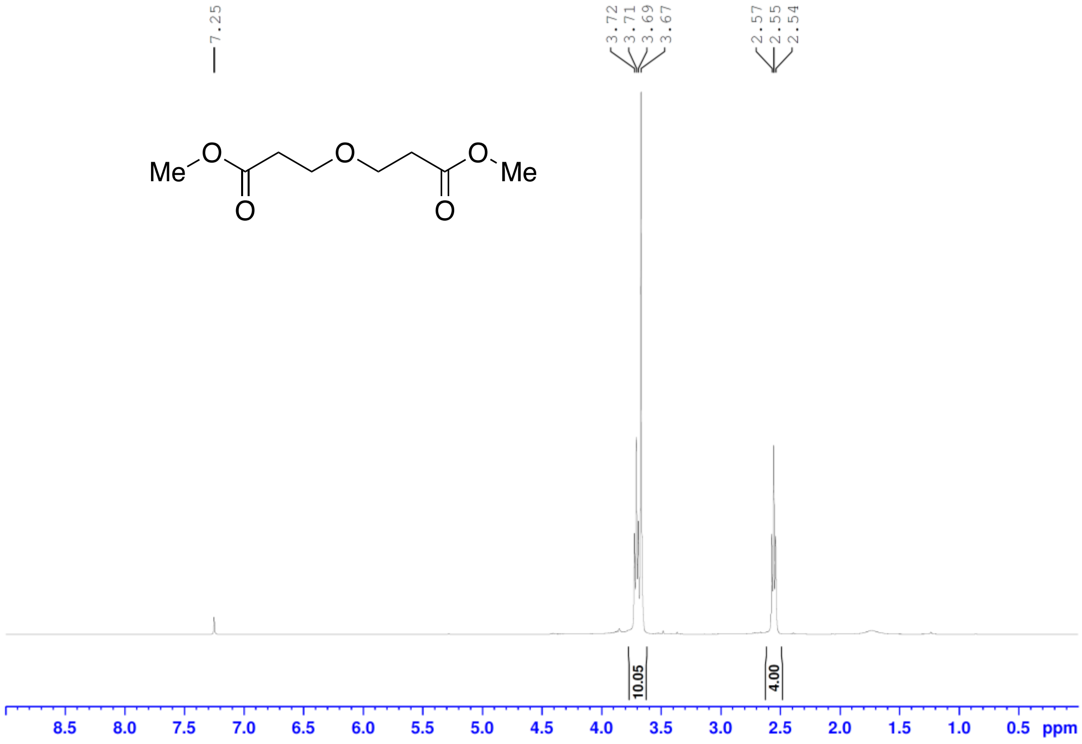

^{1}H NMR (400 MHz, $CDCl_3$) of compound **7a**

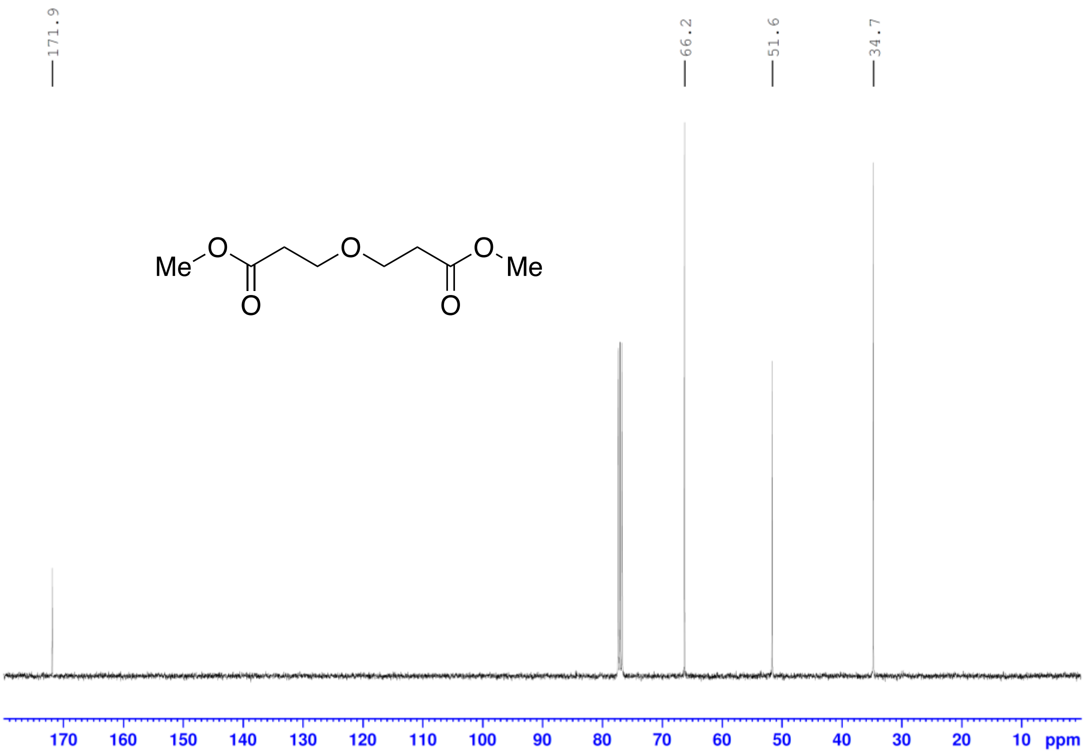

^{13}C{^{1}H} NMR (100 MHz, $CDCl_3$) of compound **7a**

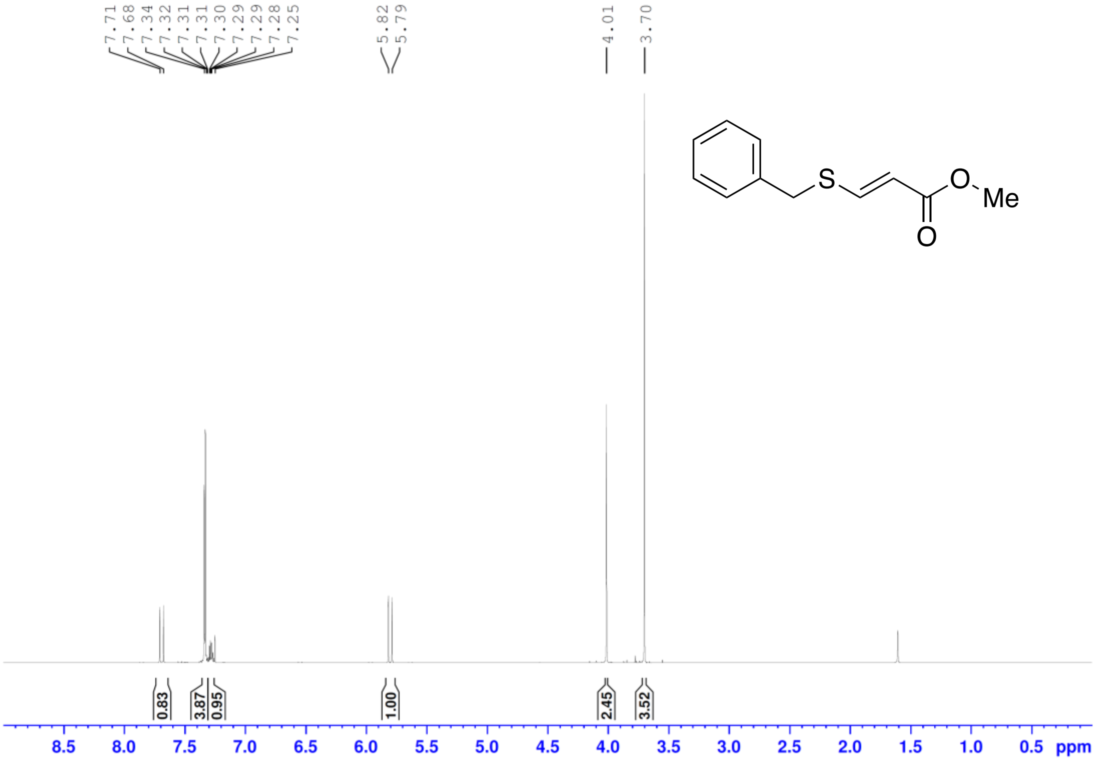

^{1}H NMR (400 MHz, $CDCl_3$) of compound **8a**

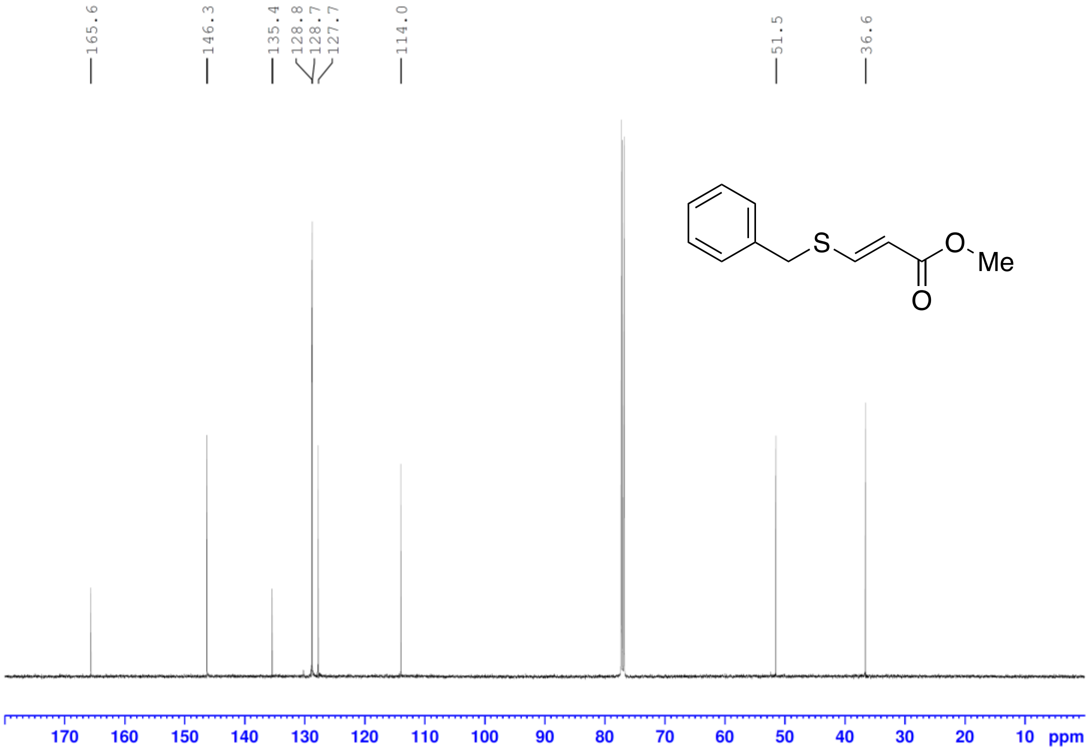

$^{13}C\{^1H\}$ NMR (100 MHz, $CDCl_3$) of compound **8a**
